# Nonsense-mediated mRNA decay and associated splicing patterns in neurodevelopmental disorders

**DOI:** 10.3389/fmolb.2026.1914793

**Published:** 2026-08-21

**Authors:** Polina Anisimova, Anastasia Filat’eva, Victor Tarabykin, Elena Kondakova

**Affiliations:** 1 Institute of Neuroscience, Lobachevsky State University of Nizhny Novgorod, Nizhny Novgorod, Russia; 2 Department of Genetics and Life Sciences, Sirius University of Science and Technology, Sochi, Russia

**Keywords:** alternative splicing (AS), neurodevelopmental disorders (NDD), nonsense-mediated mRNA decay (NMD), poison exon, premature termination codon (PTC), RBP (RNA-binding proteins)

## Abstract

Nonsense-mediated mRNA decay (NMD) is a basic post-transcriptional mechanism ensuring the fidelity of many biological processes including brain development. Together with alternative splicing, it regulates the inclusion of poison exons. NMD is involved in the control of multiple processes during brain development such as neural progenitor proliferation and differentiation, neuronal migration, axonal guidance, and synaptic plasticity. Under physiological conditions, this mechanism safeguards neuronal identity and the functional maturation of the brain. When disrupted, the consequences range from structural cerebral anomalies to cognitive impairment and epilepsy. This review examines NMD-mediated regulatory mechanisms across different stages of brain development. Special emphasis is placed on how dysfunction in NMD pathway components—specifically core degradation factors, the exon junction complex, and neuron-specific splicing regulators—underpins an extensive array of neurodevelopmental disorders (NDDs). Furthermore, we delineate the relationship between the position of a premature termination codon (PTC) within a transcript and the resulting molecular outcome. While the degradation of aberrant mRNAs often leads to haploinsufficiency, their escape from NMD might result in the accumulation of truncated proteins with dominant-negative effects, thereby causing specific clinical phenotypes in affected patients. Elucidating these mechanisms is essential for both the interpretation of variant pathogenicity and the development of targeted therapeutic strategies.

## 1 Introduction

The human central nervous system (CNS) represents a unique biological system characterised by an exceptionally diverse transcriptomic landscape (Silbereis et al., 2016; Wei et al., 2025). Alternative splicing (AS) is an evolutionarily ancient and highly conserved cellular process in which exons from the same gene are combined in various arrangements, leading to the formation of multiple related mRNA transcripts. Current estimates suggest that AS affects the expression of more than 95% of human genes (Mukherjee and Nongthomba, 2024; Su et al., 2018).

In the brain, RNA processing is critical across all stages of neuronal development—from neurogenesis and differentiation to synaptic plasticity (Schieweck et al., 2021). AS occurs with significantly greater frequency in the CNS compared to other tissues, profoundly influencing protein function and thereby neuronal development and function (Nazim, 2024). Moreover, activity-dependent RNA localisation and translation underpin synaptic plasticity, memory, axonal guidance, and regeneration (Wang D. O. et al., 2010). Collectively, the brain possesses one of the most extensive and varied RNA landscapes which require sophisticated post-transcriptional regulatory mechanisms (Liu et al., 2025).

One of the most critical post-transcriptional regulatory mechanisms, closely linked to splicing, is nonsense-mediated mRNA decay (NMD)—a pathway for mRNA degradation triggered by premature termination codons (PTCs) (Patro I. et al., 2024; Ruiz-Gutierrez et al., 2026). NMD serves a dual function: as a quality control mechanism, it protects the organism from the synthesis of potentially deleterious truncated proteins by eliminating PTC-bearing transcripts. On the other hand, it is involved in gene expression regulation, affecting approximately 5%–15% of the cell transcriptome (Kurosaki et al., 2019; Patro A. K. et al., 2024). NMD substrates can arise from genetic mutations or errors in mRNA synthesis and processing. Approximately one-third of all human inherited diseases are caused by nonsense or frameshift mutations that introduce a PTC into the transcript (Vitale et al., 2025). Classical examples include β-thalassaemia, Duchenne muscular dystrophy, and cystic fibrosis (Miller and Pearce, 2014). Around 11% of genetic disorders are linked to PTCs, with approximately 30% of congenital defects considered a result of such mutations (Mort et al., 2008; Kolakada et al., 2024).

The central nervous system seems to be particularly sensitive to NMD dysfunction. Neurological disorders are frequently observed in patients with mutations in genes encoding NMD factors. (Carrard and Lejeune, 2023). Furthermore, a systematic study revealed that of 56 genes directly or indirectly involved in the NMD mechanism, 31 (55%) are implicated in neurodevelopmental disorders (Kochinke et al., 2016). These data strongly suggest that NMD impairment serves as a convergent pathogenic mechanism in many forms of NDD, including intellectual disability, autism spectrum disorders, and epilepsy (Montazaribarforoushi and Jolly, 2025).

NMD and alternative splicing are functionally interdependent. Splicing defects can generate NMD substrates, for example, through the inclusion of “poison exons” that introduce PTCs. Conversely, NMD regulates the expression of many RNA-binding proteins (RBPs), including splicing factors, creating complex feedback loops (García-Moreno and Romão, 2020). A well-characterised mechanism involves PTBP1 protein that promotes the skipping of exon 10 in the Ptbp2 gene. This introduces a PTC and triggers NMD-mediated transcript degradation, thereby repressing PTBP2 expression in cells where PTBP1 is present (Boutz et al., 2007). Understanding how NMD and associated splicing mechanisms coordinate neuronal development, and how their dysregulation leads to disease, is paramount for the development of novel diagnostic and therapeutic strategies (Hu K. et al., 2026; McMahon and Maquat, 2025).

In this review, we examine the current state of knowledge regarding the role of the NMD pathway and its associated splicing mechanisms in the pathogenesis of neurodevelopmental disorders. Particular attention is given to the contribution of various NMD component impairments to NDD pathogenesis, including the role of NMD in non-canonical manifestations of pathogenic variants in NDD.

## 2 Fundamental mechanisms of NMD

The human genome contains approximately 20,000 protein-coding genes (Varabyou et al., 2023). The functional diversity of the proteome significantly exceeds the number of genes due to the alternative splicing (AS) mechanism. AS allows the synthesis of several protein isoforms from a single pre-mRNA template. This process significantly expands the diversity of the transcriptome and affects more than 95% of multi-exon human genes (Manuel et al., 2023; Laine and Freiberger, 2025; Jiang and Chen, 2021). Due to AS, the number of protein-coding transcripts is approximately 100,000 (Laine and Freiberger, 2025).

Alternative splicing is a regulated step in pre-mRNA maturation, during which a single primary unprocessed transcript can be read in different ways due to the variable inclusion or excision of exonic and/or intronic regions. This results in the formation of several mature mRNA isoforms from a single gene locus (Chen and Manley, 2009). Up to several dozen mRNA isoforms can be transcribed and spliced from a single human gene (Mattioli and Bulyk, 2026). However, only a fraction of them serve as a template for the synthesis of full-length functional proteins. Many transcripts contain premature stop codons (PTCs) and are subject to degradation *via* the mechanism of nonsense-mediated mRNA decay (NMD) (Zavileysky et al., 2025).

Nonsense-mediated mRNA decay is an evolutionarily conserved mechanism of mRNA quality control in eukaryotes. It ensures the degradation of abnormal transcripts containing premature stop codons or extended 3′-untranslated regions (3′UTRs), thereby preventing the synthesis of truncated and potentially toxic proteins (Carrard and Lejeune, 2023; Ottens et al., 2024; Behera et al., 2025; Pan et al., 2025; Das and Kumar Panigrahi, 2025). PTCs can arise in mRNA as a result of point nonsense mutations, insertions or deletions leading to a frameshift, as well as due to errors in pre-mRNA processing (Teodorowicz and Mühlemann, 2026; Hu K. et al., 2026). In addition, a premature stop codon can be introduced by targeted alternative splicing through the inclusion of specific poison exons (PE). PE inclusion serves as a tool for regulating transcript levels (Fair et al., 2024; Neil et al., 2025). The mechanism by which the cell uses alternative splicing in combination with NMD is abbreviated as AS-NMD (Boudreault et al., 2026).

NMD is considered one of the main mRNA quality control systems in eukaryotes (Patro I. et al., 2024). The efficiency of elimination of PTC-containing transcripts is estimated at 75%–100% (Carrard and Lejeune, 2023). Even in cases where defective mRNAs escape degradation and persist in the cytoplasm, the synthesis of potentially toxic truncated proteins is effectively blocked. The UPF1 protein (a key factor in NMD) not only triggers nucleolytic degradation of the target but also directly represses translation. Concurrently, the protein quality control system is activated: nascent aberrant peptides undergo ubiquitin-dependent cotranslational degradation in proteasomes (Kuroha et al., 2009).

NMD not only promotes transcriptome clearance of defective mRNAs but also participates in the regulation of gene expression. The AS-NMD mechanism regulates the expression level of 5%–10% of human genes, including genes encoding splicing factors and neuronal developmental proteins (Mendell et al., 2004). This mechanism, known as regulated non-productive splicing and translation, allows the cell to control the amount of functional mRNA (Mironov et al., 2023). This strategy enables a rapid and flexible genomic response to external stimuli and developmental signals, maintaining cellular homeostasis without the need to alter transcription (Choe et al., 2014; Lejeune, 2022).

Depending on the transcript architecture and the composition of protein complexes binding to mRNA, the NMD pathway can be realised through various molecular scenarios. The current model distinguishes between canonical and noncanonical NMD mechanisms, which differ in the set of initiation factors and the mode of PTC recognition (Asthana et al., 2022; Hassan et al., 2025).

### 2.1 Canonical and noncanonical NMD pathways

**FIGURE 1:**
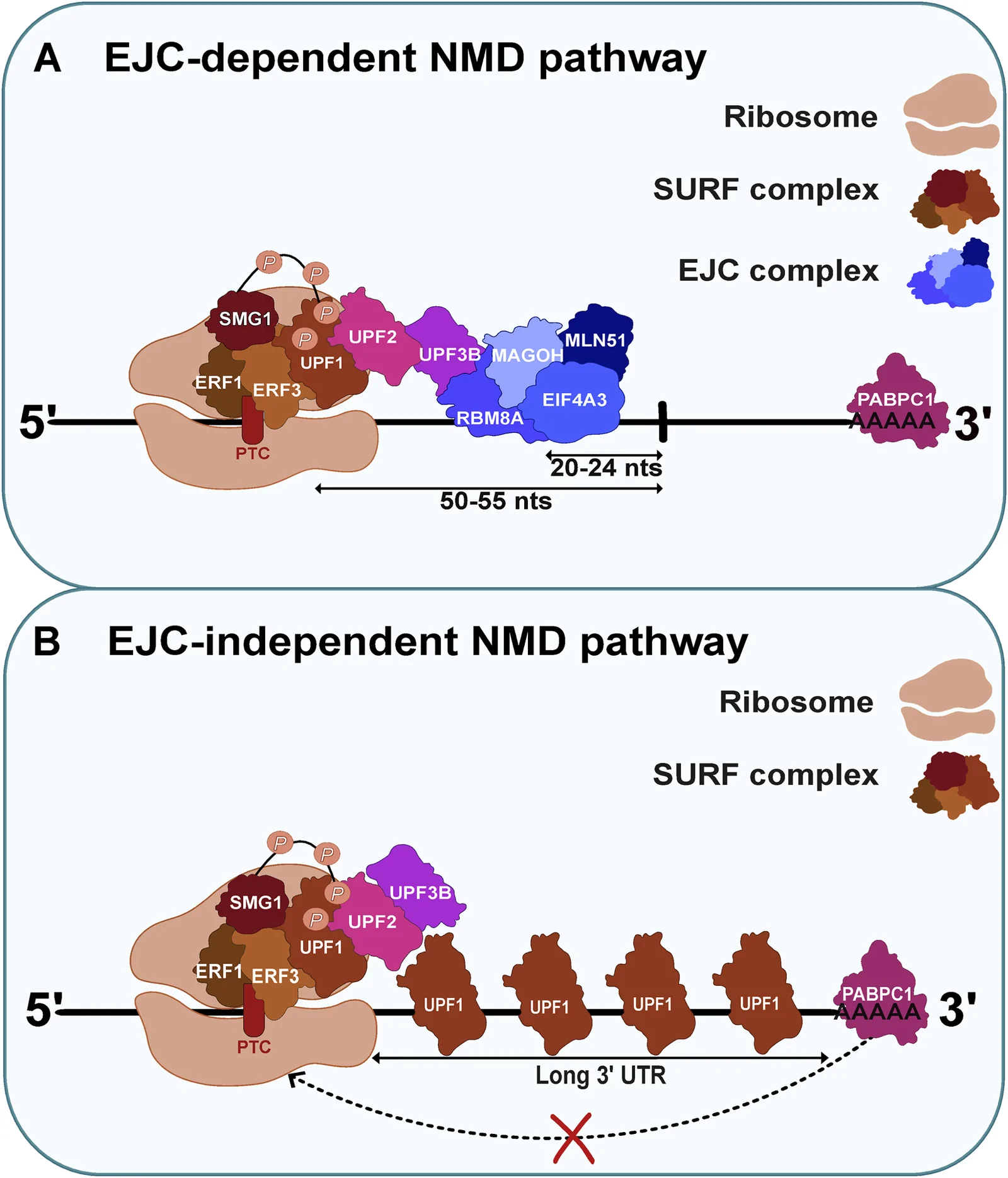
Canonical and noncanonical NMD pathways. **(A)** EJC–dependent NMD pathway. Translation termination at a premature termination codon (PTC) located more than 50–55 nucleotides upstream of the exon-exon junction (EJC) leads to the recruitment of the SURF complex (SMG1-UPF1-ERF1-ERF3). Interaction with the downstream exon-junction complex (EIF4A3-RBM8A-MAGOH-MLN51), mediated by UPF2 and UPF3B, triggers SMG1-mediated phosphorylation of UPF1 and subsequent mRNA degradation. **(B)** EJC–independent NMD pathway. In transcripts with abnormally long 3′-UTRs, the significant distance between the terminating ribosome and the PABPC1 protein prevents the transmission of the inhibitory signal from the poly(A) tail to the termination factors. The absence of this contact allows the UPF1 protein to bind unimpeded to the 3′-UTR and initiate the degradation pathway, independent of the presence of EJC positional markers.

Initiation of the canonical (EJC-dependent NMD) pathway is determined by the relative positions of the stop codon and protein splicing markers [Figure 1A]. During pre-mRNA maturation, the exon junction complex (EJC) is established 20–24 nucleotides upstream (towards the 5′end) of each exon–exon junction (Hir et al., 2000; Titus et al., 2021). It is based on four key proteins: EIF4A3, RBM8A (also known as Y14), MAGOH, and MLN51, as well as a number of additional regulatory factors (Asthana et al., 2022). EIF4A3 is the central component—an RNA helicase that directly binds the mRNA strand. RBM8A and MAGOH form an inseparable heterodimer that holds EIF4A3 to the mRNA. MLN51 further stabilises this construct, enhancing EIF4A3’s affinity for mRNA (Kim et al., 2001; Kataoka et al., 2001; Ballut et al., 2005; Ferraiuolo et al., 2004; Chazal et al., 2013; Noble and Song, 2007).

According to the canonical model, a stop codon is recognised by the control system as premature if it is located more than 50–55 nucleotides upstream of the last exon-exon junction (Nagy and Maquat, 1998; Sato and Singer, 2021). A surviving EJC downstream of the ribosome stop point serves as a signal for degradation (Sato and Singer, 2021). PTCs located in the last exon do not typically initiate NMD because they lack EJC complexes downstream (Hassan et al., 2025). EJCs act as important molecular markers that initiate canonical NMD and ensure the efficient degradation of aberrant transcripts (Kolakada et al., 2024).

The NMD mechanism is a translation-dependent process that involves multiple cellular components and is inextricably linked to translation initiation (Ishigaki et al., 2001; Maquat et al., 2010). During initiation, cap-binding complex proteins CBP80 and CBP20 coordinate the initial scanning of mRNA. At this stage, the control mechanism distinguishes premature stop codons from natural ones before translation begins at steady state (Ishigaki et al., 2001). The degradation process is triggered by ribosome collision with the PTC located upstream of the EJC (Lejeune, 2017). Premature translation termination initiates the assembly of the SURF (SMG1-UPF1-ERF1-ERF3) complex (López-Perrote et al., 2016; Causier et al., 2017). SURF then binds to UPF2, UPF3B, and EJC proteins to form the degradation-inducing complex (DECID) (Hug and Cáceres, 2014). A key step in NMD pathway activation in mammals is SURF binding to EJC, which initiates SMG1-mediated phosphorylation of the RNA helicase UPF1 (Kashima et al., 2006). This process activates UPF1, allowing it to play a central role in the subsequent degradation of transcripts with premature stop codons (Lavysh and Neu-Yilik, 2020; Boehm et al., 2025).

Phosphorylated UPF1 protein serves as a central platform for effector complex assembly. UPF1 interaction with UPF2 and UPF3B factors associated with the downstream EJC closes the regulatory chain and recruits additional proteins—SMG5, SMG6, and SMG7 (Boehm et al., 2021; Chamieh et al., 2008). It is proposed that the specific UPF1 phosphorylation pattern determines the choice of mRNA degradation pathway. Phosphorylation at residue T28 recruits the endonuclease SMG6, which makes an internal cut in the transcript near the PTC. This cut exposes the free 5′and 3′ends to exoribonucleases. Phosphorylation of S1096 site preferentially recruits the SMG5-SMG7 complex, which coordinates decapping and deadenylation (Okada-Katsuhata et al., 2012).

The UPF1 phosphorylation-dephosphorylation cycle initiates NMD. This promotes the recruitment of the decapping protein DCP2 and the deadenylating proteins PARN and CCR4. Decapping at the 5′end recruits the exoribonuclease XRN1, and deadenylation targets the transcript for the exosome complex, which degrades it in the 3'→5′direction (Wahle and Sebastiaan Winkler, 2013; Yamashita et al., 2005; Chang et al., 2019). Thus, the cell utilizes multiple degradation mechanisms to ensure the elimination of potentially dangerous mRNA molecules (Lejeune, 2022).

After transcript degradation is complete, SMG5–7 recruit protein phosphatase 2A (PP2A) to dephosphorylate UPF1. This is necessary for its release and participation in new quality control cycles (Kurosaki et al., 2014). The domain architecture and motifs of the UPF and SMG proteins are described in more detail in the reviews (Behera et al., 2025; Sun et al., 2023; Pan et al., 2025; Mailliot et al., 2022).

Non-canonical (EJC-independent) NMD is an alternative pathway of the mRNA quality control system, in which defective mRNA is recognised and destroyed without the involvement of the exon junction EJC complexes [Figure 1B]. In normal mRNA transcripts, termination occurs near the poly(A) tail, which facilitates efficient interaction between the PABPC1 protein and the termination factor ERF3. This contact ensures correct translation termination (Osawa et al., 2012; Kozlov and Gehring, 2010).

EJC-independent NMD is activated when an abnormally long distance arises between the stop codon and the PABPC1 protein. Such extended 3′-UTRs in mammals are enriched in GC-rich motifs, a configuration that promotes recruitment of the UPF1 protein (Kolakada et al., 2024; Pan et al., 2025). UPF1 then competitively binds eukaryotic release factor 3 (eRF3). (Kervestin et al., 2012). UPF1 is hyperphosphorylated by the SMG1 enzyme, which leads to the recruitment of the mRNA degradation complex and its destruction (Muñoz et al., 2023). The efficiency of EJC-independent NMD has been shown to be more sensitive to UPF2 and UPF3B levels compared to the canonical pathway, resulting in a significant change in the stability of the corresponding transcripts when they are deficient (Wallmeroth et al., 2022).

The canonical and non-canonical NMD pathways determine the molecular scenarios of mRNA degradation. However, the choice of a specific degradation pathway depends on the architecture of the molecule itself (Staszewski et al., 2023). An important natural tool for creating defective mRNAs is the existence of poison exons—genomic elements whose presence in mature mRNA predetermines its recognition by the quality control system. On the other hand, the use of poison exons to reduce mRNA stability serves as one of the mechanisms for fine-tuning post-transcriptional control of gene expression levels in healthy tissues *via* the AS-NMD pathway (Dalgliesh, et al., 2025b).

### 2.2 Contribution of poison exons to AS-NMD

**FIGURE 2:**
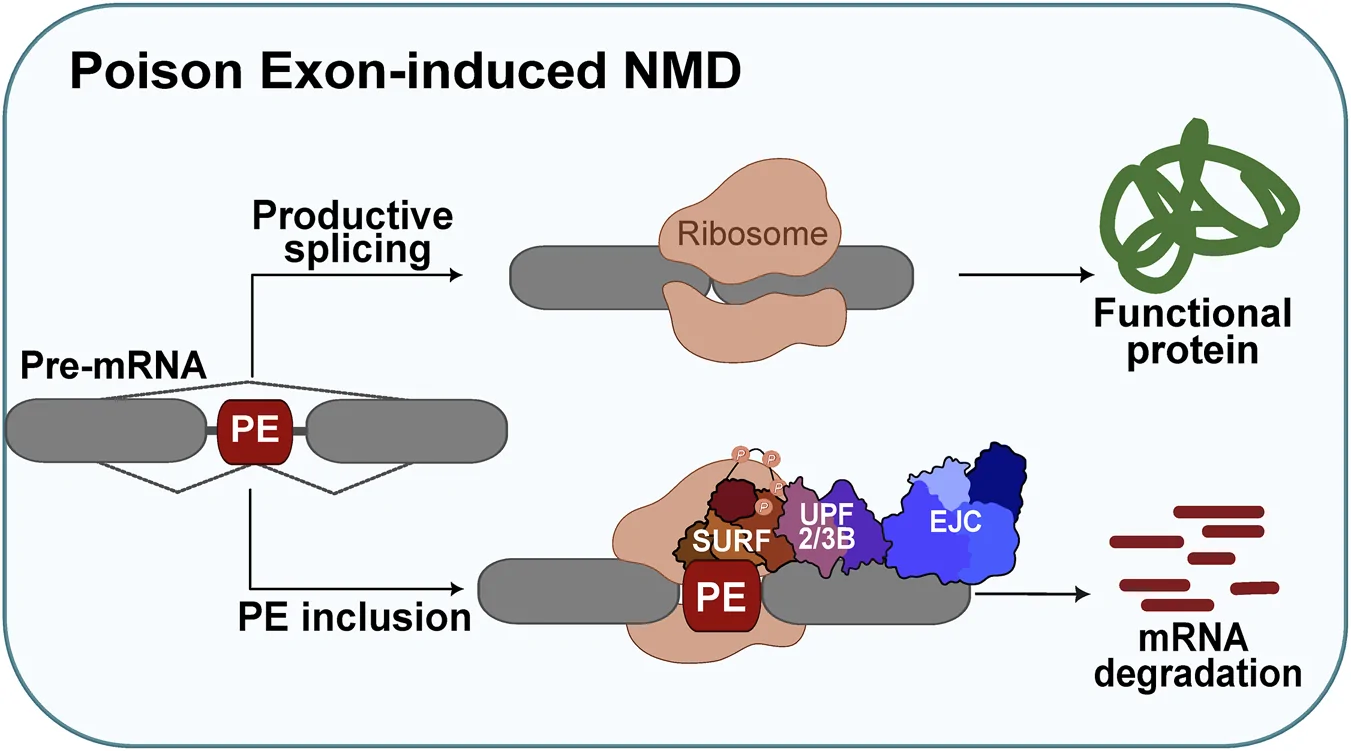
AS–NMD mechanism induced by poison exon inclusion. Alternative splicing can facilitate retention of a PTC–containing PE into the mature transcript. PE inclusion directs the mRNA to degradation through the canonical (EJC-dependent) pathway, whereas its exclusion (exon skipping) leads to productive translation of the full-length functional protein.

A poison exon is a highly conserved alternative exon that serves as a tool for regulating gene expression. Inclusion of PE introduces a PTC, which activates the NMD pathway and leads to mRNA degradation [Figure 2]. Unlike splicing errors, this is a targeted mechanism that allows for rapid reduction in protein levels without terminating transcription (Belleville et al., 2024; Neil et al., 2025; Dalgliesh et al., 2025a; Bakshi and Isom, 2025).

The biological role of the AS-NMD pathway, mediated by the inclusion of poison exons, is supported by their frequent overlap with ultraconserved genomic elements identical in humans and rodents (Saltzman et al., 2008; Titus et al., 2021; Margasyuk et al., 2024; Dalgliesh, et al., 2025b). The evolutionary fixation of these sequences is evident in the architecture of the brain transcriptome: to date, more than 1000 genes whose expression are controlled by PE-dependent mechanisms have been identified. This regulatory system enables the complex dynamics of neurogenesis, allowing for flexible adjustment of the dosage of key proteins during the development and differentiation of nervous tissue (Titus et al., 2021).

Poison exons were first discovered through bioinformatics analysis of expressed sequences aligned with the human genome. The study showed that PEs are present in ∼35% of alternative splicing events and affect approximately 23% of all extant isoforms, confirming the importance of this regulatory mechanism (Lewis et al., 2003; Carvill and Mefford, 2020).

Transcripts containing PEs are often referred to as the “dark matter” of the transcriptome (Baboo and Cook, 2014; Kapranov and Laurent, 2012). Due to their rapid degradation by the NMD system, they are difficult to detect using standard sequencing methods, even if their transcription level was initially very high (Dalgliesh, et al., 2025b). To identify such PE, functional genomics methods based on artificial suppression of the NMD system have been used. Blocking key factors (UPF1, SMG5, SMG6, SMG7) by knockdown or chemical inhibitors allows the stabilization of PE-containing transcripts, preventing their rapid degradation and making them available for analysis (Neil et al., 2025; Britto-Borges et al., 2024). In 2020, a team of scientists led by Bradley made a breakthrough in the study of PE by introducing a CRISPR/Cas9-based method called pgFARM (paired-guide RNA for alternative exon removal) (Thomas et al., 2020). This method has been generalised to measure the functional significance of poison exons on a genome-wide scale (Thomas et al., 2020; Leclair and Anczuków, 2022).

PEs are particularly important for maintaining cellular homeostasis by controlling the expression of splicing factors. These proteins do not directly repress their own transcription initiation or elongation. Instead, they activate the retention of PTC-containing exons in their own pre-mRNAs, which facilitates their subsequent degradation (Dalgliesh, et al., 2025b).

### 2.3 AS-NMD control by RNA-binding proteins

The AS-NMD mechanism directly depends on the efficient incorporation of alternative PTC-containing exons into mature mRNA. This dynamic process is coordinated by a complex network of trans-acting factors. Among these factors, families of RNA-binding proteins (RBPs) play a key role, recognising cis-regulatory sequences in pre-mRNA (Jeong et al., 2025). The interaction of these factors with RNA determines not only the tissue-specific splicing profile but also the accuracy of RNA quality control systems, which requires a detailed examination of key groups of regulatory proteins.

Key players in this process include the SR and hnRNP families, as well as tissue-specific regulators represented by the RBFOX, PTB, and NOVA families (Fisher and Feng, 2022). By forming dynamic regulatory networks, these proteins ensure splicing specificity at different stages of development and in various cell types. Understanding how this network functions requires a detailed analysis of each family, starting with the best-studied classical splicing factors—SR proteins.

#### 2.3.1 Serine/arginine-rich proteins

The family of serine-arginine-rich proteins (SR proteins) includes 14 major members: SRSF1–12, as well as TRA2A and TRA2B that share a common evolutionary origin (Long and Caceres, 2009; Leclair et al., 2020). PEs in SR protein genes correspond to ultraconserved genomic sequences and are fundamental for the viability of the organism (Dalgliesh, et al., 2025b). A characteristic feature of SR proteins is the presence of one or two N-terminal RNA recognition domains (RRM) and one C-terminal arginine/serine-rich domain (RS). The RS domain consists of at least 50 amino acids with an RS content of >40% (Manley and Krainer, 2010; Jin, 2022). The RRM domain is involved in pre-mRNA binding, and RS is required for controlling protein-protein interactions during spliceosome assembly (Wagner and Frye, 2021; Cascarina and Ross, 2022).

**FIGURE 3:**
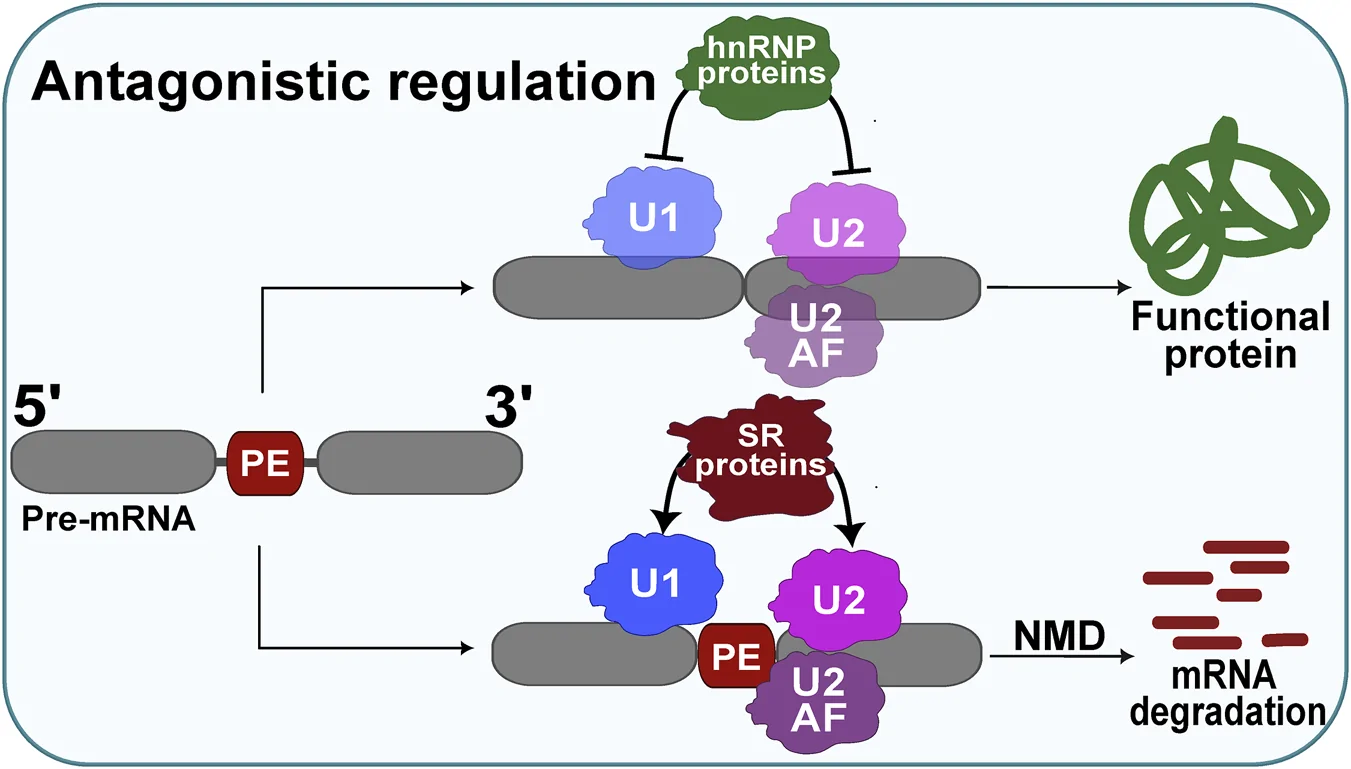
Regulation of AS-NMD by splicing factors. The poison exon inclusion or exclusion is determined by antagonistic splicing factors. Typically, hnRNP proteins act as repressors, inhibiting PE recognition by the spliceosome (U1, U2, U2AF) and promoting functional protein synthesis. In contrast, SR proteins act as activators, facilitating PE inclusion, thereby reducing gene expression *via* NMD.

By participating in constitutive and alternative splicing, SR proteins coordinate its initial stages, ensuring correct exon recognition (Park et al., 2019; Vorobeva et al., 2024). By binding to exonic (ESE) and intronic (ISE) enhancers (cis-regulatory elements), SR proteins form complexes that stabilize the binding of U1 snRNP to the 5′splice site and U2AF to the 3′splice site. Through such protein-protein interactions, SR factors effectively mark exon boundaries, facilitating their inclusion into mature mRNA even in the presence of weak canonical splicing signals [Figure 3] (Sapir and Reiner, 2023). In addition, SR proteins are involved in a number of off-splicing processes: mRNA export and degradation, as well as translation regulation (Leclair et al., 2020; Slišković et al., 2022). Thus, fluctuations in SR protein levels are capable of altering the expression of a vast network of target genes and alternative splicing patterns.

PE-mediated autoregulation of SR protein expression acts *via* a negative feedback loop (Pervouchine et al., 2019). This ensures rapid and efficient changes in the level of SR proteins themselves, allowing the cell to promptly adjust their concentration in response to transcriptional changes (Dalgliesh, et al., 2025b). When SR proteins are in excess, they stimulate the incorporation of PE into their own pre-mRNAs, which subsequently leads to their nonsense-mediated decay (Dalgliesh, et al., 2025b; Jangi and Sharp, 2014; Dalgliesh, et al., 2025a; Leclair et al., 2020; Grosse et al., 2021). SR proteins are able to directly interact with transcripts of other SR proteins, suggesting widespread cross-regulation of each other’s expression *via* AS-NMD (Lareau et al., 2007).

#### 2.3.2 Heterogeneous nuclear ribonucleoproteins

The second important class of trans-regulatory factors are heterogeneous nuclear ribonucleoproteins (hnRNPs), a family of 20 canonical multifunctional RNA-binding proteins (HNRNPA1 to HNRNPU) (Tilliole et al., 2024; Schorr and Mangone, 2021; Bampton et al., 2020; Sapir and Reiner, 2023). hnRNP proteins are characterised by the presence of several evolutionarily conserved RNA-binding domains (RBDs), which are connected by flexible linker regions. With the exception of HNRNPU, these proteins have a tandem domain organization, which allows them to simultaneously interact with different RNA segments (Brandão-Teles et al., 2024; Bampton et al., 2020). The most significant and evolutionarily conserved ones include RNA recognition domains (RRM), K-homologous domains (KH), and glycine-arginine-rich fragments (GAR/RGG boxes) (Jiang et al., 2023; Low et al., 2021). By interacting with other RNA-binding proteins, hnRNPs form complexes that regulate all stages of mRNA maturation and metabolism, including splicing. Since key RNA maturation processes occur within the nucleus, most hnRNPs contain nuclear localization signals (NLSs), which ensure their preferential accumulation in the nucleoplasm (Nakielny and Dreyfuss, 1996; Bampton et al., 2020).

While hnRNPs are traditionally considered functional antagonists of SR proteins, the nature of this interaction goes beyond direct competition for binding (Vorobeva et al., 2024; Slišković et al., 2022). While SR proteins interact with exonic and intronic enhancers (ESE/ISE), facilitating exon inclusion, hnRNPs bind to silencers (ESS/ISS), blocking access of spliceosome components (Slišković et al., 2022; Talukdar et al., 2011; Lee and Rio, 2015). The final choice of splice sites is determined by the balance of these factors and is concentration-dependent: local predominance of SR proteins neutralizes the effect of repressors and prevents exon skipping (kyung Oh et al., 2013; Jang et al., 2014; Kędzierska and Piekiełko-Witkowska, 2017). It is important to note that such regulation is often combinatorial and is maintained through complex cross-talk within the hnRNP family (Sapir and Reiner, 2023). Moreover, the level of hnRNP proteins themselves in the cell is often controlled by an autoregulatory mechanism through the inclusion of poison exons, which trigger NMD when they are in excess (Lin J. Q. et al., 2023; Rossbach et al., 2009; Xu et al., 2019; Kemmerer et al., 2018). hnRNP proteins are key regulators of constitutive and alternative splicing in cells. By ensuring the accuracy of mRNA maturation, they determine the course of processes critically necessary for normal tissue development (Brandão-Teles et al., 2024).

#### 2.3.3 RNA-binding Fox-1 homologues

The RNA-Binding Fox-1 homologues (RBFOX) gene family (RBFOX1/A2BP1, RBFOX2/RBM9, RBFOX3/NeuN) encodes evolutionarily conserved (from *Caenorhabditis elegans* to humans) RNA-binding proteins that play a key role in regulating alternative splicing of thousands of transcripts, primarily in nervous and muscle tissues (Qiu et al., 2026; Shen J. et al., 2024). A distinctive feature of these factors is the presence of a highly conserved RNA recognition domain (RRM) that specifically binds to the (U)GCAUG motif (Mukherjee and Nongthomba, 2024). Such binding sites are localized at important regulatory points of the transcriptome: in pre-mRNA introns, 3′-untranslated regions (3′-UTR) of mature mRNAs, and hairpin structures of pre-microRNA. RBFOX proteins exhibit position-dependent effects: binding to motifs within ∼200 nucleotides of a downstream intron stimulates inclusion of an alternative exon, while interaction with the exon itself or the upstream intronic region promotes its skipping (Gehman et al., 2011; Tomassoni-Ardori et al., 2025).

RBFOX proteins exhibit dual intracellular localization. They are predominantly found in the nucleus, where they regulate pre-mRNA splicing. Some isoforms can be expressed in the cytoplasm, where they regulate RNA stability and translation (Begg et al., 2020; Peyda et al., 2025). In the nucleus, a significant portion of RBFOX is associated with the Large Assembly of Splicing Regulators (LASR), a multiprotein complex that recognizes additional RNA motifs. LASR comprises eight RNA-binding factors (HNRNPM, HNRNPH/F, HNRNPC, DDX5, NF-110, HNRNPUL2, NF-45, Matrin3), and, in some cases, MECP2 (Damianov et al., 2016). Tandem tyrosine residues within the intrinsically disordered C-terminal domain (CTD) of RBFOX are important for the assembly of RBFOX/LASR into higher-order complexes and splicing activation (Ying et al., 2017; Peyda et al., 2025; Tomassoni-Ardori et al., 2025). It was established that RBFOX is required for the recruitment of LASR to the target RNA through interaction with the (U)GCAUG motif. Formation of this complex stabilizes the association of RBFOX with RNA, stimulating its interaction with nearby GCAUG sites (Peyda et al., 2025).

CLIP-seq analysis data indicate that RBFOX family proteins are capable of interacting not only with the canonical (U)GCAUG motif, but also with the related GCACG sequence (Lambert et al., 2014; Begg et al., 2020). This interaction may be mediated by indirect recruitment of the SUP-12 or HNRNPM factors within the LASR complex. This allows RBFOX to exhibit splicing enhancer activity even in the absence of primary (U)GCAUG sites (Conboy, 2017; Damianov et al., 2016; Begg et al., 2020). It was found that with an increase in the intracellular concentration of RBFOX proteins, they begin to bind to secondary motifs with lower affinity. A similar increase in RBFOX expression is characteristic of differentiating neurons, providing an additional wave of alternative splicing regulation at the expense of previously inactive sites (Begg et al., 2020).

Expression of RBFOX family genes is controlled by a mechanism of auto- and cross-regulation. All three mammalian paralogs are capable of binding to the (U)GCAUG motif in introns flanking the coding exon of the RRM domain. This interaction induces exon skipping and the synthesis of a dominant-negative isoform. This isoform lacks RNA binding ability but antagonizes the activity of the full-length protein, suppressing its function as a splicing enhancer (Conboy, 2017).

Another important mechanism for maintaining RBFOX homeostasis is AS-NMD. Inclusion of conserved poison exons leads to a frameshift and subsequent transcript degradation (Damianov and Black, 2010).

Rbfox3, in addition to stimulating the skipping of Rbfox2 exon 6, enhances the inclusion of two cryptic exons, 5* and 6*, both of which introduce a PTC into Rbfox2 mRNA. Inhibition of the NMD pathway leads to the additional accumulation of these transcripts, confirming their degradation *via* NMD (Dredge and Jensen, 2011).

#### 2.3.4 Polypyrimidine tract-binding proteins

The Polypyrimidine Tract-Binding proteins (*PTBP)* gene family (PTBP1, PTBP2, PTBP3) encodes proteins that bind predominantly to poly-pyrimidine-rich regions of RNA through four RNA recognition motifs. PTBP1 (HNRPI) also belongs to the ubiquitously expressed heterogeneous nuclear ribonucleoprotein (hnRNP) subfamily. Members of this family share more than 70% amino acid homology (Oberstrass et al., 2005). *PTBP1* is ubiquitously expressed. *PTBP2* (also known as neuronal PTB - nPTB) is tissue-specific in the nervous system, while *PTBP3* is expressed in immune cells (Zhu et al., 2020; Dawicki-McKenna et al., 2023).

PTBP1 is a post-transcriptional regulator that controls splicing, translation, stability, and localization of mRNA (Takahashi et al., 2015). Its expression is controlled at the transcriptional level by other proteins through alternative splicing: the presence of exon 11 in the transcript promotes the synthesis of a functional product (Méreau et al., 2015).

PTBP1 plays the most important role in the growth and differentiation of neural cells, functioning throughout neurogenesis (Yap et al., 2012; Zheng et al., 2012; Linares et al., 2015; Vuong et al., 2016; Zhang X. et al., 2016). In NSCs, the PTBP1 protein functions as a repressor of neuronal splicing, blocking premature differentiation. PTBP2 represses a set of alternative exons that are prevalent in the adult brain, preventing them from being included prematurely in the embryonic brain. The key mechanism is realized through the regulation of the poison exon 10 (PE10) in the PTBP2 transcript. PTBP1 binds to the intronic regions around PE10, stimulating its inclusion and subsequent degradation of the PTBP2 mRNA *via* the NMD pathway (Keppetipola et al., 2012). High levels of PTBP1 block the expression of the neuronal factor PTBP2, keeping cells in a proliferative state. Decreasing the level of PTBP1 is a necessary trigger for the initiation of neurogenesis (Raj and Blencowe, 2015; Li et al., 2014; Ling et al., 2016).

PTBP1 controls through AS-NMD the expression of hundreds of genes critical for neurogenesis. In particular, the protein regulates the key postsynaptic gene Psd-95. PTBP1 and PTBP2 repress the splicing of exon 18, introducing PTC and triggering NMD. The sequential loss of PTBP1 and then PTBP2 during embryonic development ensures the inclusion of exon 18 and triggers the synthesis of PSD-95 (Zheng et al., 2012). Additionally, PTBP1 controls neuronal survival through PE in the Bak1 gene. The reduction of PTBP1 in immature neurons allows the inclusion of neuronal-specific microexon 5, which triggers NMD and inhibits the synthesis of the pro-apoptotic protein BAK1 (Lin L. et al., 2020).

Thus, when the neural progenitors enter the differentiation stage, the level of PTBP1 decreases, which releases the PTBP2 transcript from NMD control and initiates the neuronal maturation program (Pina et al., 2022).

#### 2.3.5 Neuro-oncological ventral antigens

The *NOVA1* and *NOVA2* genes (neuro-oncological ventral antigens 1 and 2) have 75% sequence identity and encode neuron-specific RNA-binding proteins with three K-homologous (KH) domains. KHs recognize the YCAY motif on pre-mRNA and regulate alternative splicing (Piton, 2025; Tajima et al., 2023). NOVA factors have been shown to independently regulate more than 700 alternative splicing events *in vivo* (Ule et al., 2006). In addition, it has been shown that NOVA1 and NOVA2 modulate alternative polyadenylation, a post-transcriptional process in which different mRNAs with different 3′-untranslated regions are produced from individual genes (Licht et al., 2019; Hu W. et al., 2014). NOVA factors also regulate the retention of introns by acting as a cis-acting scaffold for the AS PTBP2 factor (Saito et al., 2019). NOVA proteins function as position-dependent PE switches. Binding of PTBP1/NOVA above PE during transcription prevents its activation, and below PE, on the contrary, stimulates its activation, triggering the NMD mechanism (Linares et al., 2015; Ule et al., 2006).

NOVA1 in humans is more strongly expressed in the cerebellum, whereas NOVA2 is predominantly expressed in cortical structures (Jelen et al., 2010; Piton, 2025). In the brain, the average expression of the NOVA1 gene is approximately three times higher than the expression of NOVA2 (Meldolesi, 2020). NOVA2 plays an important role in the development of laminar structure, motor coordination, and synaptic formation through the regulation of alternative splicing of transcripts that are critical for these processes (Saito et al., 2019). In addition to post-transcriptional modifications, NOVA2 is required for long-term potentiation mediated by the GIRK2 kinase channel and the GABA(B) receptor in CA1 pyramidal neurons (Huang C. S. et al., 2005). NOVA2 is also an RBP that promotes the biogenesis of neuronal circular RNA (circRNA) in the developing brain (Knupp et al., 2021).

## 3 The role of AS-NMD in the nervous system

The mammalian brain exhibits a profound degree of transcriptomic diversity and the most complex patterns of alternative splicing compared to any other tissue in the organism (Siletti et al., 2023). All stages of neuronal development, encompassing proliferation, differentiation, neuronal migration, axonal and dendritic arborisation, as well as synaptic differentiation and plasticity, necessitate the precise spatiotemporal regulation of gene expression (Jiang and Nardelli, 2016; Patro A. K. et al., 2024; Zahr et al., 2019). NMD serves a dual function within these processes. Firstly, this pathway provides quality control by preventing the translation of defective mRNAs. Secondly, the AS-NMD mechanism regulates the quantity of synthesised protein, which is a requisite for coordinating gene expression according to the developmental stage of the neuron (Hu et al., 2026). Mutations or functional inactivation of NMD components in neurons precipitate the accumulation of toxic truncated proteins or the dysregulation of transcripts containing poison exons (Montazaribarforoushi and Jolly, 2025). Such perturbations compromise neuronal cytoskeletal architecture and dendritic branching, whilst promoting an imbalance between excitatory and inhibitory synapses, thereby culminating in severe neurodevelopmental disorders (NDDs) (Gatto, 2010).

**FIGURE 4:**
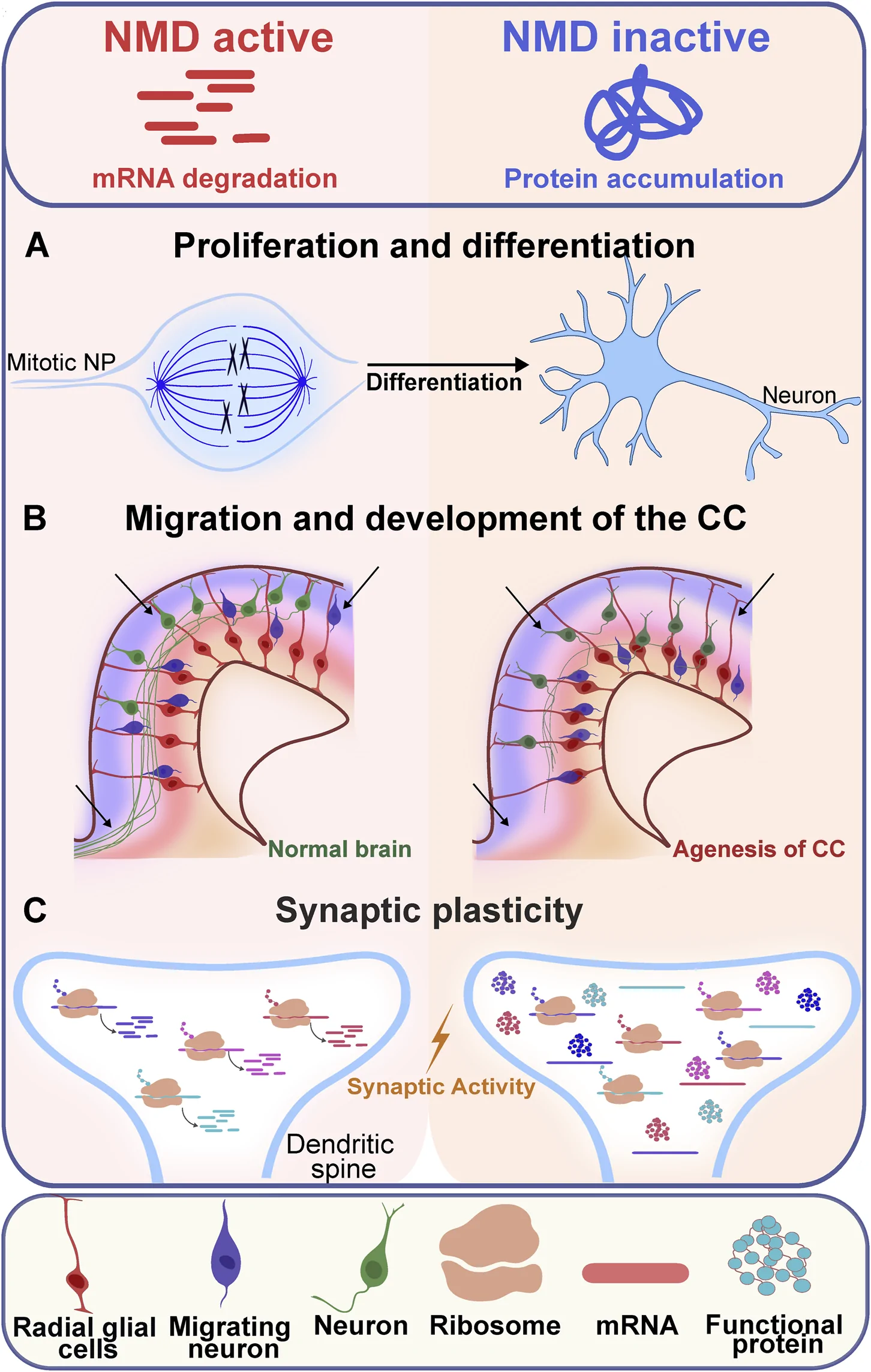
The physiological role of NMD in neurogenesis and synaptic plasticity. **(A)** Proliferation and differentiation. Active NMD (left) maintains neural progenitor cells (NPCs) in a mitotic state *via* the systematic degradation of proneural transcripts. Conversely, the inactivation of NMD (right) stabilises these pivotal transcripts, inducing protein accumulation and facilitating the transition of neural progenitors into mature, differentiated neurons. **(B)** Under physiological conditions (left), NMD coordinates both the radial migration of newly generated neurons and the navigation of commissural axons crossing the midline to form the corpus callosum (CC). The disruption of NMD (right) results in neuronal migration defects and aberrant commissural axon navigation, culminating in agenesis of the corpus callosum (ACC). **(C)** Synaptic plasticity. The role of NMD in mediating synaptic plasticity consists of the stringent spatiotemporal restriction of effector protein synthesis. This averts their unregulated accumulation within dendrites and ensures the high fidelity of the local synaptic response.

The ontogeny of the nervous system is predicated upon a delicate equilibrium between the proliferation of neural progenitor cells and their subsequent differentiation [Figure 4A]. NMD acts as a pivotal regulator in maintaining this balance, facilitating the exit of progenitor cells from the proliferative state(Lou et al., 2014; Zhuravskaya et al., 2024; Papadimitriou and Thomaidou, 2024). It has been established that during *in vitro* neurogenesis, a systematic downregulation of NMD activity occurs within neural progenitor cells; this constitutes a necessary and sufficient condition for initiating the differentiation phase (Tan and Wilkinson, 2025; Notaras et al., 2020; Zou et al., 2015). The attenuation of NMD activity stabilises mRNAs encoding proneural factors in progenitor cells, thereby promoting neuronal differentiation (Tan and Wilkinson, 2025; Zhuravskaya et al., 2024; Jaffrey and Wilkinson, 2018). Furthermore, NMD is essential for the survival of the neural progenitor population within the cerebral cortex (Lin L. et al., 2024; Tan and Wilkinson, 2025). A deficiency in NMD factors leads to cell cycle aberrations and arrest at various phases (Azzalin and Lingner, 2006; Lou et al., 2014; Lin L. et al., 2024). An alternative hypothesis posits that the abrogation of NMD induces widespread cellular toxicity, which consequently suppresses proliferation as a secondary effect (Han X. et al., 2018; Lykke-Andersen and Jensen, 2015). Consequently, dysfunctions within the NMD machinery can result in the premature differentiation of progenitor cells and the subsequent depletion of the proliferative pool. The clinical significance of NMD is underscored by the clinical presentation of microcephaly, autism spectrum disorders, and cognitive impairments in patients, which manifest as a consequence of disruptions to the aforementioned processes (Lou et al., 2014; Zou et al., 2015; Jolly et al., 2013).

The phases of proliferation and differentiation are succeeded by an equally critical stage of corticogenesis: neuronal migration, which is indispensable for appropriate cortical cytoarchitecture [Figure 4B] (Hippenmeyer, 2014; Mubuchi et al., 2024; Hansen and Hippenmeyer, 2020). The timely transition of molecular programmes, which enables neurons to alter their motility and adhesive properties at precise developmental windows, is a prerequisite for the successful assembly of the six-layered cortex (Devreotes and Horwitz, 2015; Hatanaka et al., 2016; Hansen and Hippenmeyer, 2020). However, until recently, the role of NMD in this context remained unexplored (Lin L. et al., 2026). Only recently has a study employing *in utero* electroporation and transcriptomics elucidated that NMD is an indispensable post-transcriptional regulatory mechanism governing appropriate neuronal positioning and cerebral cortical lamination (Lin L. et al., 2026).

In addition to orchestrating neuronal migration, the NMD system exerts post-transcriptional control over the guidance of commissural axons across the midline. This mechanism is predicated on the tightly regulated synthesis of the ROBO3.2 receptor within the neuronal growth cone. In neurons, ROBO3 exists in two splicing isoforms distinguished by their C-terminal intracellular domains. Robo3.1 is expressed before the midline crossing, whereas Robo3.2 is present on axons after they have traversed the commissure. The ROBO3.2 protein enhances the activity of other ROBO receptors, ROBO1 and ROBO2, which mediate the chemorepulsive response to SLIT ligands presented at the midline (Chen Z. et al., 2008). While axons migrate toward the midline, Robo3.2 mRNA accumulates in RNA granules in a translationally repressed state. Once the axon reaches the midline, cell-extrinsic midline factors trigger the translation of Robo3.2 mRNA, presumably *via* its release from the granules. The translation of Robo3.2 mRNA simultaneously triggers its degradation through the NMD pathway, thereby ensuring a transient surge in ROBO3.2 protein production to augment the function of ROBO1 and ROBO2 and repel the axon from the midline (Kurosaki et al., 2019; Chen Z. et al., 2008; Gavrish et al., 2024; Jung H. et al., 2014; Jung J. et al., 2023; Lejeune, 2022; Andreassi et al., 2018; Jaffrey and Wilkinson, 2018). The dysfunction of this mechanism leads to the aberrant accumulation of ROBO3.2, excessive axonal repulsion before the midline cross, and profound architectural abnormalities within the corpus callosum (Kurosaki et al., 2019; Perrone-Capano et al., 2021).

NMD is catalytically active not solely within the neuronal soma, but also in the most distal compartments, namely, dendrites and axons (Perrone-Capano et al., 2021). This spatial arrangement permits the localisation of mRNAs to specific subcellular domains, enabling the initiation of their translation in response to external stimuli in a stringently controlled manner (Jung H. et al., 2014; Jung J. et al., 2023; Wong et al., 2017). Such spatiotemporal activation of NMD facilitates the execution of fundamental neuronal functions at considerable distances from the cell body (Lennox et al., 2018; Howe and Patani, 2023; Jaffrey and Wilkinson, 2018; Engel et al., 2020).

NMD functions as a pivotal regulator of neuronal homeostasis, modulating developmental processes as well as learning and memory. NMD is considered an evolutionarily conserved mechanism that influences synaptic events (Jaffrey and Wilkinson, 2018). Its role in facilitating synaptic plasticity resides in the stringent spatial and temporal restriction of effector protein synthesis, thereby averting their unchecked accumulation within dendrites [Figure 4C] (Notaras et al., 2020; Jaffrey and Wilkinson, 2018). For instance, following the transient activation of the Arc gene, its mRNA is translocated to dendrites and translated. Subsequently, owing to the presence of introns within the 3′-UTR, it is recognised as an NMD substrate and undergoes rapid degradation (Lyford et al., 1995; Farris et al., 2014). Consequently, the Arc protein is synthesised exclusively during periods of neuronal activation. This mechanism guarantees the potentiation of synaptic transmission whilst preventing excitotoxicity, a balance that is critical for memory consolidation (Tan and Wilkinson, 2025). Furthermore, it has been demonstrated that NMD is requisite for the local regulation of GLUR1 (glutamate receptor 1) *via* the degradation of ARC and PRKAG3 transcripts (Notaras et al., 2020).

The UPF2 protein (a core component of the NMD pathway) is essential for the maintenance of dendritic spine density, synaptic plasticity, late-phase long-term potentiation (L-LTP), and cognitive function (Notaras et al., 2020). Its targeted deletion in adult neurons precipitates the attenuation of L-LTP, the onset of cognitive deficits, and an exacerbation of perseverative behaviour (Notaras et al., 2020; Asthana et al., 2022). In *Upf3b* knockout models, a deficit in dendritic spine maturation is observed within cortical pyramidal neurons (L Huang et al., 2018). Conversely, EIF4A3 functions as an endogenous brake on synaptic strength; its knockdown significantly amplifies AMPA receptor-mediated miniature excitatory postsynaptic currents (mEPSCs) in cortical neurons (Giorgi et al., 2007).

Consequently, the AS-NMD mechanism serves as an indispensable regulator at every pivotal stage of corticogenesis, and is equally requisite for the maintenance of neuronal homeostasis. This pathway is implicated in the fine-tuning of synaptic plasticity *via* the stringent spatial restriction of local protein synthesis within dendrites.

Mutations in genes encoding core AS-NMD protein factors inevitably disrupt transcriptomic equilibrium, thereby precipitating the pathogenesis of NDDs. Given that synaptic plasticity underpins higher cognitive and behavioural functions, any perturbations in the regulatory mechanisms governing synaptic protein dosage directly contribute to the aetiology of autism spectrum disorders (ASD), intellectual disability, and epilepsy (Yang R. et al., 2023).

The subsequent section provides a comprehensive analysis of the most prominent genes within the NMD machinery and their associated pathogenic variants, which delineate the clinical phenotypes of these disorders.

## 4 The contribution of NMD component impairments to the pathogenesis of NDD

### 4.1 Impairments in NMD complex components (NMD-pathies)

For an extended period, NMD dysfunction was regarded merely as a secondary effect within the context of other genetic disorders. However, recent clinical and genetic studies have demonstrated that pathogenic variants in genes encoding components of the NMD and Exon Junction Complex (EJC) give rise to distinct, monogenic forms of neurodevelopmental disorders (NDD) (Zou et al., 2015; Mefford et al., 2008). These manifest as intellectual disability (ID), autism spectrum disorder (ASD), behavioural disturbances, and speech impairments, among others. This process is driven by the fact that NMD serves not only as a quality control mechanism for mRNA containing premature termination codons (PTCs) but also as a regulator of the expression of numerous normal transcripts that are critical for neuronal development (Pervouchine et al., 2019). Depending on the functional role of the defective proteins, these impairments can be categorised into defects of core degradation factors (UPF and SMG) and defects of EJC components.

#### 4.1.1 Impairments of core degradation factors

Proteins of the UPF and SMG families are the direct executors of mRNA degradation. Mutations in the genes encoding these proteins typically result in a spectrum of severe neurodevelopmental disorders, where intellectual disability (ID) is the primary symptom (Tümer et al., 2025; Johnson et al., 2019; Mailliot et al., 2022).

##### 4.1.1.1 The UPF family (UPF1, UPF2, UPF3B)

In patients with *de novo* heterozygous *UPF1* variants, intellectual disability, motor and speech delays, and behavioural disturbances (ASD, ADHD, and aggression) are observed (Zahir et al., 2017; Tümer et al., 2025; Nakato et al., 2024). A complete loss of UPF1 function is incompatible with life, as a total knockout in mice results in early embryonic lethality (Hwang and Maquat, 2011).

In mice, the knockout of *Upf2* prolongs the cell cycle of progenitor cells and leads to a reduction in upper-layer neurons, resulting in microcephaly (Lin L. et al., 2024). In mature postsynaptic neurons, UPF2 deficiency reduces the surface expression of AMPA receptors and leads to dendritic spine immaturity. At the molecular level, this explains impairments in long-term potentiation and memory formation (Notaras et al., 2020; L Huang et al., 2018). In behavioural experiments, *Upf2*-knockout mice exhibit an ASD phenotype, including social deficits and abnormal ultrasonic vocalisations. These findings align with clinical observations in humans, as *UPF2* variants cause ASD, cognitive deficits, and pronounced speech impairments (Johnson et al., 2019).

Loss-of-function (LoF) variants in the *UPF3B* gene are associated with severe forms of X-linked intellectual disability, ASD, and early-onset schizophrenia (Nomura et al., 2025; Laumonnier et al., 2010; Jolly et al., 2013; Tarpey et al., 2007; Bufton et al., 2022). Transcriptomic analysis revealed that targets of UPF3B-dependent NMD include the mRNA of the ROBO1 and NRCAM genes (which govern axonal guidance) and ARHGAP24 (a Rho-GTPase controlling branching) (Nguyen et al., 2012; Deka et al., 2021). The loss of UPF3B leads to the aberrant accumulation of these transcripts, which morphologically manifests as reduced axonal length and pathological dendritic branching (Huang et al., 2018).

##### 4.1.1.2 The SMG family (SMG5, SMG8, SMG9).

SMG5 (Suppressor of Morphological Defect on Genitalia 5). The SMG5 protein forms a heterodimer with SMG7 and performs a dual function in the NMD system: it attracts decapping and deadenylating factors to phosphorylated UPF1 and ensures the full endonucleolytic activity of the SMG6 enzyme, without which the degradation of aberrant transcripts is significantly slowed down (Boehm et al., 2021). A patient with craniofacial dysmorphia, developmental delay, severe growth retardation, and relative macrocephaly was identified to have a homozygous synonymous variant in the *SMG5* gene, affecting the last base of the penultimate exon and disrupting canonical splicing, resulting in a decrease in the level of the full-length SMG5 protein to ∼25% of the normal (Tibbe et al., 2026). Analysis of the patient’s fibroblasts revealed an increase in cell size, Golgi apparatus, and nucleus, as well as impaired proliferation and delayed cell cycle. Transcriptome analysis demonstrated global dysregulation with activation of nucleosome assembly genes and suppression of differentiation genes, as well as global accumulation of NMD-sensitive transcripts, including an experimentally confirmed increase in the level of natural PTC-containing transcripts of the splicing factors SRSF2, SRSF4, SRSF6, and SF3B3 (Tibbe et al., 2026). In a mouse conditional knockout model of *Smg5*, early embryonic lethality was observed up to the E13.5 stage upon complete gene deletion, whereas knockout of *Smg5* in mouse embryonic stem cells did not impair their viability and proliferation, but was critically required for differentiation, which is consistent with the developmental defects observed in the patient (Chen C. et al., 2024).

SMG8 and SMG9 form a regulatory heterodimer that, as described in Section 2.1, controls the SMG1 kinase and ensures the phosphorylation of UPF1 (Kueckelmann et al., 2026; Rahikkala et al., 2022). Pathogenic variants in the *SMG8* gene, including frameshifts, disrupt the G-like and KID domains, destabilising the SMG1-SMG8-SMG9 regulatory kinase complex. This, in turn, induces pathological alterations in the phosphorylation level of UPF1 and a breakdown of transcriptomic balance (Alzahrani et al., 2020). Clinically, *SMG8* deficiency causes severe neurodevelopmental impairments, including global developmental delay, ID, microcephaly, and brain white matter anomalies (Al-Kasbi et al., 2022; Abdel-Salam et al., 2022). Additionally, patients present with facial dysmorphisms, congenital heart defects, cataracts, and short stature—a phenotype characteristic of Alazahrani-Kuwahara syndrome (ALKUS) (Abdel-Salam et al., 2022).

SMG9 is a regulatory cofactor that, upon binding to the SMG1 kinase, executes quality control of transcripts by selectively degrading them (Rahikkala et al., 2022; Lai et al., 2024). Studies on a mouse model with a complete *Smg9* knockout (*Smg9* ^−/−^) revealed early embryonic lethality. Homozygous embryos at stages E15.5 and E18.5 exhibit pronounced developmental delay, generalised oedema, haemorrhages, and other malformations. Detailed micro-CT analysis of the embryos demonstrates severe central nervous system anomalies (exencephaly, midbrain and hindbrain hypoplasia) and poor development of the visual apparatus (microphthalmia or complete anophthalmia) (Shaheen et al., 2016). In patients with NDD, biallelic pathogenic missense variants and frameshift mutations leading to premature protein truncation have been identified. The phenotype includes mild to moderate ID, dyspraxia, intentional tremor, growth retardation, microcephaly, congenital heart defects, and specific ocular anomalies (cataract, strabismus) (Altuwaijri et al., 2021; Rahikkala et al., 2022; Yang Q. et al., 2022).

#### 4.1.2 Impairments in EJC components

The EJC serves as an obligatory molecular marker that signals the position of exon-exon junctions to the NMD system and initiates the degradation of aberrant transcripts (Gehring et al., 2005). Genetic defects in the core components of the EJC block the recognition of premature termination codons, leading to the accumulation of toxic mRNAs. In the developing brain, such failure in mRNA quality control disrupts the division of neural stem cells and neural crest cells, ultimately resulting in NDD (Zou et al., 2015).

##### 4.1.2.1 EIF4A3 (eukaryotic translation initiation factor 4A3)

Autosomal recessive variants in the *EIF4A3* gene cause Richieri-Costa-Pereira syndrome, characterised by severe acrofacial dysostosis, microcephaly, and ID (Bertola et al., 2018). In mouse models and human cortical organoids, eIF4a3 haploinsufficiency has been shown to cause cell cycle arrest, specifically by lengthening mitosis in progenitor cells (Miller E. E. et al., 2017; Lupan et al., 2023). This results in the accumulation of DNA damage and the activation of massive p53-dependent apoptosis. In the early stages of neurogenesis (E13.5), cell death disrupts the formation of deep cortical layers (TBR1^+^ and CTIP2^+^ neurons), whereas at stage E16.5, the deficit of upper-layer neurons is further exacerbated by p53-independent premature differentiation of the remaining stem cells (Lupan et al., 2023). Such rapid depletion of the neural progenitor pool leads to thinning of the cerebral cortex, which phenotypically accounts for the development of severe microcephaly (Mao et al., 2017).

##### 4.1.2.2 RBM8A (RNA-binding protein 8A/Y14)

Pathogenic variants in the *RBM8A* gene are associated with autism spectrum disorders, schizophrenia, and severe TAR syndrome (thrombocytopenia with absent radius) (Boussion et al., 2020). Conditional brain knockout models (*Rbm8a* cKO) have proven that this protein is essential for maintaining the neural stem cell (NSC) pool: its haploinsufficiency triggers cell cycle arrest, p53-dependent apoptosis, and premature differentiation of progenitors in the subventricular zone. Conversely, *Rbm8a* overexpression exerts the opposite effect, blocking the exit of NSCs from the cell cycle and artificially maintaining them in a hyperproliferative state at the expense of neurogenesis (Zou et al., 2015; Wang L. et al., 2023). Thus, RBM8A acts as a dose-dependent switch of cellular fate, whose transcriptomic imbalance leads to the disorganisation of cortical layers and the development of severe microcephaly (McSweeney et al., 2020; Mao et al., 2015).

### 4.2 Dysregulation of poison exons

#### 4.2.1 Impairments in trans-regulation: RNA-binding proteins

Splicing mechanisms are determined by both cis-elements (sequences within the RNA molecule itself) and trans-regulation—external factors that recognise these sequences and govern the process. At the level of post-transcriptional processing, trans-regulation is predominantly executed by RNA-binding proteins (RBPs) (Zhuravskaya et al., 2024). These proteins control the inclusion and exclusion of poison exons (PEs) in neuronal transcripts. Their dysfunction leads to a shift in PE/AS-NMD switches and, consequently, to impaired expression of multiple genes critical for the development and maturation of nervous tissue (Huang et al., 2025; Zhuravskaya et al., 2024).

##### 4.2.1.1 PTBP family (PTBP1, PTBP2)

In patients with *de novo PTBP1 (hnRNP1)* variants, a syndrome involving skeletal dysplasia, dysmorphism, and neurodevelopmental impairments has been described. Start-loss mutations lead to impaired nuclear localisation of the protein and its excessive retention in cytoplasmic P-bodies (Masson et al., 2025).

Homozygous *Ptbp1* knockout in mice leads to lethality between stages E8.5 and E12.5. Ptbp1 deficiency causes a sharp decrease in proliferative activity due to cell cycle arrest in the G2/M phase, as well as massive apoptosis (Suckale et al., 2011).

In patients with heterozygous *de novo PTBP2* variants, intellectual disability, ASD, and severe speech impairments are observed. Similar to *PTBP1*, these mutations cause cytoplasmic retention of the protein and its accumulation in P-bodies, pointing to a common pathogenic mechanism within the PTBP family (Masson et al., 2025).

Systemic *Ptbp2* knockout in mice results in neonatal lethality due to respiratory failure. Local knockout in the forebrain causes postnatal degeneration of the neocortex and hippocampus, with death occurring by the third week of life (Li et al., 2014; Licatalosi et al., 2012).

##### 4.2.1.2 RBFOX family (RBFOX1, RBFOX2, RBFOX3)

Pathogenic variants of the *RBFOX1* gene are clinically associated with ASD, intellectual disability, and epilepsy (W.-W. Zhao, 2013).

Experimental models have demonstrated that *Rbfox1* mediates cell-type-specific alternative splicing programmes in cortical interneurons, regulating their integration into developing cortical circuits (Wamsley et al., 2018). The disruption of autoregulation of its own PE exon 19 in pathological *Rbfox1* variants renders protein levels uncontrollable and disorganises the splicing of neuronal circuits (Lee J.-A. et al., 2016). *Rbfox1* knockout in mice increases susceptibility to seizures and induces ASD-like behaviour (O’Leary et al., 2022).

*Rbfox2* mutations in mice are associated with cerebellar ataxia, cerebellar hypoplasia, delayed motor development, and intellectual disability (Gehman et al., 2012).

Patients with variants in the *RBFOX3* (*NeuN*) gene have been described to have delayed cognitive development, severe speech impairments, and epileptic seizures (Wang H.-Y. et al., 2015).

*Rbfox3* knockout mice are viable but show increased seizure susceptibility, spatial memory deficits, and increased anxiety (Wang H.-Y. et al., 2015).

Triple knockout of *Rbfox 1/2/3* exhibits defects in the alternative splicing of cytoskeletal, membrane, and synaptic proteins, with impairment of neuronal electrophysiological activity (Jacko et al., 2018).

##### 4.2.1.3 NOVA family (NOVA1, NOVA2)

*De novo* pathogenic variants in *NOVA2* result in severe NDD, comprising ID, motor and speech delays, ASD, hypotonia, spasticity, ataxic gait, and brain MRI anomalies. At the molecular level, pathogenic variants in the central region of the gene, affecting the final KH RNA-binding domain, reduce the affinity of NOVA2 for the YCAY motif and impair its function (Engal et al., 2024). Consequently, neurite outgrowth and axonal guidance are impaired, which is consistent with the mapping of *Nova2* binding sites in the mouse genome primarily within genes related to synaptic function and axonal guidance, such as *Dcc*, *Robo2*, *Slit2*, and *Epha5* (Forti et al., 2025).

Since synaptic protein transcripts are specific PE targets for NOVA, double *Nova1/2* knockout in mice leads to spontaneous generalised seizures. These molecular changes are associated with altered PE inclusion/exclusion in synaptic transcripts; for instance, increased PE inclusion in Dlg3 (with a corresponding decrease in mRNA levels) and decreased PE inclusion in Scn9a (with an increase in mRNA levels) (Carvill and Mefford, 2020). In mice, homozygous deletion of the *Nova1* gene results in early postnatal lethality due to abnormal motor function (Tajima et al., 2025).

##### 4.2.1.4 SR-related splicing factor (SRSF1, SRRM2, SRRM4)

SRSF1 (ASF/SF2) protein is a prototypical SR protein that regulates constitutive and alternative splicing (Paul et al., 2023). In 17 patients with heterozygous *SRSF1* variants, a phenotype was identified that included developmental delay and intellectual disability, hypotonia, neurobehavioral disorders, and variable skeletal (66.7%) and cardiac (46%) abnormalities (Bogaert et al., 2023).

Its complete loss in mice leads to fetal mortality (Paul et al., 2023). Functional studies in *Drosophila* and DNA epigenetic analysis confirmed that *SRSF1* haploinsufficiency causes a syndromic neurodevelopmental disorder due to a partial loss of SRSF1-mediated splicing activity (Paul et al., 2023). Conditional knockout of Srsf1 in the developing cortex (Srsf1cKO) leads to massive p53-dependent apoptosis of neural progenitors in the ventricular zone and a severe depletion of the NPC pool (Weber et al., 2023; Yu et al., 2022).

The SRRM2 (SRm300) protein belongs to the SR-related splicing factor family and functions as a structural scaffold for nuclear speckles and a component of the catalytic spliceosome complex, facilitating the interaction between mRNA and the splicing machinery (Cuinat et al., 2022; Zhang M. et al., 2024). In a cohort of 22 patients with variants of loss of function in *SRRM2* (12 frameshifts, 8 nonsense variants, two microdeletions), the clinical picture included developmental delay with mild intellectual disability, mainly speech disorders, autistic traits or ADHD, generalized hypotension, overweight, and dysmorphic facial features (Cuinat et al., 2022; Shen X. et al., 2025).

Heterozygous Srrm2 ± mice exhibit large-scale changes in gene expression of synaptic function and mitochondrial metabolism, decreased SynGAP levels in synapses, pronounced deficiency of oligodendrocytes and myelination (especially in the striatum), impaired start reflex and carotid spindle deficiency, which resembles changes in schizophrenia (Lin L. et al., 2026).

The *SRRM4* gene encodes the neuron-specific protein SRRM4 (nSR100), a master regulator of alternative splicing of neuronal microexons 3–27 nucleotides long (Irimia et al., 2014; Quesnel-Vallières et al., 2015). Pathogenic *de novo* variants of the splicing site at position +2 of the donor site of intron 5 (c.399+2T>C and c.399+2T>G) are described in patients with multisymptom developmental disorder, hyperkinetic syndrome of early infancy, dystonia and chorea. Functional studies revealed aberrant processing of SRRM4 pre-mRNA and subsequent splicing disorders in dozens of target genes (Harrer et al., 2026).

Mice heterozygous for exons 7 and 8 deletion (Srrm4+/Δ7-8) reproduce the pattern of impaired splicing of microexons characteristic of ASD and demonstrate an autism-like phenotype (aversion to conspecifics, altered density of dendritic spines, impaired synaptic transmission). Whereas a complete knockout leads to gross disorders of neurite growth, disorganization of cortical stratification and skipping of microexons in genes of the cytoskeleton and axonal navigation (Quesnel-Vallières et al., 2015; Harrer et al., 2026).

##### 4.2.1.5 ARGLU1 (arginine and glutamate rich 1)

The ARGLU1 protein is a key mediator that couples transcription and alternative splicing through direct interaction with the MED1 subunit of the Mediator complex (Zhang D. et al., 2011). The level of its own protein ARGLU1 is controlled by the mechanism of autoregulatory AS-NMD: in its excess, splicing is shifted towards an isoform containing an alternative exon within the ultra-conserved element (UCE), which introduces PTC and directs the transcript to degradation *via* the NMD pathway (Pirnie et al., 2017). Thus, *ARGLU1* is an example in which the dysfunction of a single RBP simultaneously triggers the autoregulation of its own transcript through UCE-dependent PE and NMD-mediated degradation of the target gene transcript (Yao et al., 2023; Pirnie et al., 2017).

The clinical significance of this mechanism is supported by the identification of pathogenic *ARGLU1* variants in patients with intellectual disability and autism spectrum disorders (Jin S. C. et al., 2020; Hiatt, Thompson, Prokop, Lawlor, Gray, Bebin, Rinne, Kempers, Pfundt, Van Bon, et al., 2019).

A complete knockout of *Arglu1* in mice leads to embryonic lethality at the E9.5 stage, while a knockdown in zebrafish causes neurodevelopmental disorders and heart defects (Dehghani-Tafti et al., 2019). Conditional knockout of Arglu1 in the developing mouse cortex causes radial glial cells to detach from the ventricular zone, prolongs the mitosis of progenitors, accumulates p53, and induces apoptosis, which collectively leads to microcephaly (Yao et al., 2023). The central molecular mechanism is the aberrant splicing of Mdm2 and Mdm4 transcripts, which are key negative regulators of p53. The skipping of exon 3 of Mdm2 eliminates the p53-binding domain, while the skipping of exon 5 of Mdm4 causes a frameshift and degradation of the transcript through the NMD pathway, which collectively relieves the inhibition of p53. Deletion of p53 largely eliminates this phenotype (Yao et al., 2023).

#### 4.2.2 Impairments in cis-regulation: Expression switches

Section 4.2.1 examines cases where the PE/AS-NMD system is functional, yet a pathogenic variant disrupts a specific cis-regulatory element, causing the spatiotemporal switch to fail. The genes discussed in this chapter are unified by the fact that PE/AS-NMD serves as a normal regulatory tool for fine-tuning expression in neurons. Pathogenic variants in these genes do not disrupt the global splicing machinery but rather break a single switch in a single gene—which is sufficient to cause severe NDD.

##### 4.2.2.1 FLNA (filamin A)

*FLNA* encodes the actin-binding protein filamin A, which provides a mechanical link between the actin cytoskeleton and signalling proteins. It also regulates cellular migration processes (Zhang Y. et al., 2026). Under normal conditions, the *FLNA* gene contains a PE that is skipped in NPCs but included in neurons, where it introduces a PTC and triggers NMD (Zhang X. et al., 2016).

Sequencing of patients with Periventricular Nodular Heterotopia (PVNH) revealed a pathogenic variant in *FLNA* that disrupts the cis-regulatory element required for interaction with the PTBP1 protein. Functionally, this results in the stimulation of aberrant PE inclusion in NPCs, leading to a partial loss of FLNA function and an atypical PVNH syndrome. It is noteworthy that this variant is located within a deep intronic region and is not detectable *via* standard exome sequencing (Zhang X. et al., 2016). This highlights the necessity of expanding genetic testing to include flanking non-coding elements, which play a crucial role in neurological disorders.

##### 4.2.2.2 SYNGAP1 (synaptic Ras GTPase-Activating Protein 1)

The SYNGAP protein localises to the postsynaptic density of excitatory neurons and, through its GAP-activating and structural scaffolding functions, regulates RAS/RAP signalling pathways and AMPA receptor trafficking while maintaining the excitation/inhibition balance (Zhang J. et al., 2026). SYNGAP1-related neurodevelopmental disorder (SRD) is a monogenic condition caused by heterozygous LoF mutations in the *SYNGAP1* gene; the phenotype includes global developmental delay, ID, epilepsy, ASD, and behavioural disturbances (Zhang J. et al., 2026; Treccarichi et al., 2026). The high transcript degradation during early neurogenesis gives way to stabilization during neuronal maturation, ensuring high levels of SYNGAP1 protein during the period of active synaptogenesis (Yang R. et al., 2023).

In patients with ID and autistic features, the variant c.1676 + 5 G>A almost entirely disrupted the donor splice site and caused retention of intron 10, whereas the variant c.1677-2_1685del disrupted the canonical acceptor site of intron 10 and significantly increased A3SS usage (Yang R. et al., 2023). A splice-switching oligonucleotide (SSO) was developed that converted the non-productive SYNGAP1 isoform into the functional form in human iPSC-derived neurons, partially restoring SYNGAP1 expression. Consequently, *SYNGAP1* A3SS represents a promising therapeutic target: SSO-mediated blockade of this switch does not require correction of the coding mutation and is potentially applicable to a broad range of patients with SYNGAP1 haploinsufficiency, regardless of the specific pathogenic variant (Dawicki-McKenna et al., 2023).

##### 4.2.2.3 ROBO3 (Roundabout guidance receptor 3)

The clinical consequences of disrupting this mechanism are well characterised in horizontal gaze palsy with progressive scoliosis (HGPPS). HGPPS is defined by a congenital or early-onset absence of conjugate horizontal eye movements and progressive scoliosis. The disorder results from defects in the axonal crossing of the midline by specific neuronal populations in the rhombencephalon and potentially the spinal cord (Günbey et al., 2024). ID, hypergonadotropic hypogonadism, hearing impairment, and transient motor disorders are additional clinical features. Here, NMD acts as a molecular timer for axonal guidance associated with translation in the distal compartment of the neuron. Disruption of this mechanism destroys the topography of commissural pathways and produces the HGPPS phenotype (Pinero-Pinto et al., 2020).

In mice, the *Robo3.2* transcript contains an intron retention that introduces a PTC in a new reading frame, making it an NMD target; however, the transcript can also produce the Robo3.2 protein with an alternative C-terminus. Because Robo3.2 is translationally repressed until it reaches the midline, it evades NMD. After the axon crosses the midline, local signals trigger a transient increase in Robo3.2 levels, and this transcript concurrently degrades *via* NMD. This provides precise spatiotemporal control of protein expression (Howe and Patani, 2023).

##### 4.2.2.4 *SCN family genes (SCN1A, SCN8A*)

The SCN1A (NAV1.1) and SCN8A (NAV1.6) proteins together account for over 95% of sodium channel transcripts in the brain and are responsible for the majority of known neurological sodium channelopathies (Meisler et al., 2021). A poison exon (exon 20N), situated between canonical exons 20 and 21, has been described in the *SCN1A* gene. In the normal fetal brain, the inclusion level of this PE is high, which maintains low NAV1.1 expression. As neurons mature, a splicing “switch” occurs: the poison exon begins to be skipped, leading to a sharp increase in the level of full-length NAV1.1 protein (Voskobiynyk et al., 2021).

The pathogenesis of severe myoclonic epilepsy of infancy (Dravet syndrome) is directly linked to an imbalance in this process. Deep intronic mutations or splice site variants that enhance exon 20N inclusion result in excessive mRNA degradation *via* the NMD pathway. *Scn1a* ^*+*^ */KI* mice exhibit up to a 50% reduction in *Scn1a* mRNA and Nav1.1 protein in the brain, along with features observed in other mouse models of Dravet syndrome, including premature death, seizures, and hyperactivity (Voskobiynyk et al., 2021).

Additionally, two more PEs were identified in introns 1 and 22 of *SCN1A*, the inclusion of which is also regulated during human brain development (Tang et al., 2025). It has been demonstrated that the Targeted Augmentation of Nuclear Gene Output (TANGO) method—based on antisense oligonucleotide (ASO) blockade of non-productive splicing in *Scn1a*—restores *Scn1a* expression in a mouse model of Dravet syndrome, reducing the number of seizures and the frequency of sudden unexpected death in epilepsy (SUDEP) (Han Z. et al., 2020). A similar regulatory mechanism has been discovered in the *SCN8A* gene, where a poison exon (exon 18N or 20N, depending on the transcript) controls the expression threshold of the NAV1.6 channel (Plummer et al., 1997). Exon 18N contains multiple stop codons and is normally included in 5%–10% of transcripts in the adult human brain (Heighway et al., 2022). However, under certain pathological conditions, the inclusion level of the poison exon rises sharply. This activates NMD-dependent decay and leads to a reduction in NAV1.6 channel density, which severely impairs the initiation of action potentials in the axon initial segment (Liang et al., 2021).

Table 1 provides a systematic classification of NMD pathway genes and associated regulators of alternative splicing and expression. This categorisation is based on their functional roles in post-transcriptional regulation. For each gene, specific molecular characteristics and the corresponding clinical phenotypes are outlined.

**TABLE 1:** Genes of the NMD system: molecular functions and clinical manifestations.

Defects in components of the core NMD complex (NMDopathies)			
	Gene	Molecular function	Patient phenotype
Impairments in NMD complex components (NMD-pathies)	*UPF1*	ATP-dependent RNA helicase (Staszewski et al., 2023)	Intellectual disability (ID) Motor and speech delay; Behavioural disturbances (ASD, ADHD, aggression). (Zahir et al., 2017)
	*UPF2*	UPF3B and UPF1 binding (Szeto et al., 2025)	Autism spectrum disorder (ASD); Cognitive deficit; Pronounced speech impairments. (Johnson et al., 2019)
	*UPF3B*	Binding to EJC components and recruitment of the UPF2 factor (Tarpey et al., 2007; Laumonnier et al., 2010)	Severe forms of X-linked ID; ASD; Early-onset schizophrenia. (Laumonnier et al., 2010)
	*SMG5*	forming a heterodimer with SMG7, and coordinating the decapping and deadenylation of aberrant transcripts (Boehm et al., 2021)	Craniofacial dysmorphia; Severe growth retardation; Relative macrocephaly (Tibbe et al., 2026)
	*SMG8*	Formation of a regulatory heterodimer that controls SMG1 kinase and ensures UPF1 phosphorylation (Kueckelmann et al., 2026; Rahikkala et al., 2022)	Global developmental delay; ID; Microcephaly; White matter anomalies. (Al-Kasbi et al., 2022)
	*SMG9*		Mild to moderate ID; Dyspraxia; Intentional tremor; Microcephaly. (Rahikkala et al., 2022)
EJC Components	*EIF4A3*	Central EJC RNA helicase; links the EJC to the transcriptome for subsequent NMD (Lupan et al., 2023)	Richieri-Costa-Pereira syndrome (acrofacial dysostosis, microcephaly, and ID). (Bertola et al., 2018)
	*RBM8A*	Formation of an obligate heterodimer with the MAGOH factor, which anchors the EIF4A3 helicase to the mRNA molecule (Hir et al., 2016; Ishigaki et al., 2013)	ASD; Schizophrenia; Microcephaly(Boussion et al., 2020)
Dysregulation of poison exons			
Trans-regulation: RNA-binding proteins (RBPs)	*PTBP1*	Repressor of neuron-specific alternative exons in immature cells (Keppetipola et al., 2012)	Syndrome with skeletal dysplasia, dysmorphism, and neurodevelopmental impairments. (Masson et al., 2025)
	*PTBP2*	Repressor of alternative exons enriched in the adult brain to prevent premature inclusion in the embryonic brain (Pina et al., 2022)	ID; ASD; Severe speech impairments. (Masson et al., 2025)
	*RBFOX 1*	RNA-binding proteins; activators and repressors of alternative exons *via* the (U)GCAUG motif (Damianov and Black, 2010)	ASD; ID; Epilepsy. (Wamsley et al., 2018)
	*RBFOX 2*		Cerebellar ataxia; Cerebellar hypoplasia; Motor delay; ID. (Gehman et al., 2012)
	*RBFOX 3*		Cognitive developmental delay; Pronounced speech delay; Less commonly, epileptic seizures. (Lal et al., 2013)
	*NOVA1*	RNA-binding proteins recognising the YCAY motif on pre-mRNA, primarily in the ventral brain and spinal cord (Piton, 2025)	ID; Motor delay; Speech impairments; Autistic traits; Corpus callosum hypoplasia. (Piton, 2025)
	*NOVA2*	RNA-binding proteins recognising the YCAY motif on pre-mRNA, primarily in the cortex, hippocampus, and cerebellum (Jelen et al., 2010)	ID; Motor and speech delay; ASD; Hypotonia; Spasticity; Ataxic gait; Corpus callosum hypoplasia. (Engal et al., 2024)
	*SRSF1*	Key SR protein; activates exon inclusion through ESE/ISE binding (Paul et al., 2023)	ID; Hypotonia; Neurobehavioral disorders; Variable skeletal (66.7%) and cardiac (46%) abnormalities (Bogaert et al., 2023)
	*SRRM2*	the structural framework of nuclear specks and a component of the catalytic spliceosome complex, facilitating the interaction between mRNA and the splicing machine machinery (Cuinat et al., 2022; Zhang M. et al., 2024)	ID; Mainly speech disorders, Autistic traits or ADHD; Generalized hypotension, overweight; Dysmorphic facial features (Cuinat et al., 2022; Shen X. et al., 2025).
	*SRRM4*	Master regulator of alternative splicing of neuronal microexons 3–27 nucleotides long (Irimia et al., 2014; Quesnel-Vallières et al., 2015)	Hyperkinetic syndrome of early infancy; Dystonia and chorea (Quesnel-Vallières et al., 2015; Harrer et al., 2026)
	*ARGLU1*	Mediator of transcription and alternative splicing through interaction with the MED1 subunit of the Mediator complex (Zhang D. et al., 2011)	ID; ASD; Microcephaly (Yao et al., 2023)
Cis-regulation: Expression switches	*FLNA*	Actin-crosslinking protein; crosslinks F-actin filaments *via* the ABD domain and 24 immunoglobulin-like repeats (Zhang Y. et al., 2026)	Periventricular nodular heterotopia (PVNH). (Zhang X. et al., 2016)
	*SYNGAP1*	Regulates Ras/Rap signalling pathways and AMPA receptor trafficking (Zhang J. et al., 2026)	SYNGAP1-related neurodevelopmental disorder (SRD) (global developmental delay, ID, epilepsy, ASD, and behavioural disturbances). (Zhang J. et al., 2026)
	*ROBO3*	Transmembrane receptor of the Roundabout family with an extracellular domain (Basha et al., 2023)	Horizontal gaze palsy with progressive scoliosis (HGPPS) – an autosomal recessive disorder associated with impaired axonal guidance. (Günbey et al., 2024; Pinero-Pinto et al., 2020)
	*SCN1A*	Nav1.1 sodium channel subunit (Meisler et al., 2021)	Dravet syndrome (Han Z. et al., 2020)
	*SCN8A*	Nav1.6 sodium channel subunit (Meisler et al., 2021)	Epileptic encephalopathy; ID; Ataxia. (Meisler et al., 2021)

Despite the shared molecular underpinnings associated with mRNA degradation, the clinical manifestations of the described impairments are remarkably diverse. The systematisation of these data illustrates how disruptions to different links within the same pathway lead to specific phenotypes. Even within a single family (e.g., RBFOX or NOVA) or a single protein complex (UPF/SMG), a deficiency in individual components results in distinct forms of NDD, which is dictated by their unique spatiotemporal roles in brain development.

## 5 Non-canonical pathogenic mechanisms in NDD

### 5.1 Genes in which pathogenicity is mediated *via* NMD-Induced haploinsufficiency

Although NMD evolved as a protective mechanism to prevent the accumulation of potentially toxic truncated proteins, its activity can become pathogenic in the context of dosage-sensitive genes (Yang L. et al., 2024). In such instances, the very efficiency of the quality control system becomes the critical factor that converts a heterozygous mutation into a state of functional haploinsufficiency. Below are the most representative examples of genes for which the contribution of NMD-dependent transcript degradation to the pathogenesis of NDD has been experimentally proven. It is noteworthy that this list is continuously expanding as data accumulate; therefore, we have also included several candidate genes of interest for further study.

#### 5.1.1 ARID1B (AT-rich interaction domain 1B)

The ARID1B protein is a large subunit of the BAF (BRG1/BRM-associated factor) chromatin-remodelling complex, which is an evolutionarily conserved analogue of the yeast SWI/SNF complex (Moffat et al., 2022). ARID1B serves as the DNA-binding subunit of this complex and regulates chromatin accessibility for transcription factors. Evidence suggests that the BAF complex is involved in regulating the exit of neural progenitors from the cell cycle and their differentiation into postmitotic neurons (Sim et al., 2015; Pagliaroli et al., 2021).

Pathogenic *ARID1B* variants are among the most frequent causes of non-syndromic intellectual disability and Coffin–Siris syndrome (CSS) (Lian et al., 2020). An *Arid1b* ^*+/−*^ mouse model exhibits reduced corpus callosum and dentate gyrus, decreased cortical thickness, and diminished proliferative activity (Lu et al., 2021). The vast majority of described pathogenic *ARID1B* variants are nonsense mutations or small insertions/deletions that result in a frameshift and the introduction of a PTC. Studies confirm that such transcripts undergo effective NMD-mediated degradation, leading to a twofold reduction in *ARID1B* protein levels (Lu et al., 2021). ARID1B haploinsufficiency disrupts the balance between proliferation and differentiation of neural stem cells, as the BAF complex loses its ability to open access to the promoters of neurogenesis genes. This leads to microcephaly, hypoplasia of the corpus callosum, and cognitive developmental delay (Moffat et al., 2022; Sofronova et al., 2022).

#### 5.1.2 WDR26 (WD repeat domain 26).

This gene encodes an adapter protein that is a component of the CTLH E3 ubiquitin ligase complex, which regulates the cell cycle and signalling in neurons (Onea et al., 2025).

The central pathogenic mechanism is NMD-mediated haploinsufficiency. Analysis of mRNA stability in lymphoblastoid cell lines from patients with frameshift variants revealed a significant reduction in cDNA levels and direct evidence of transcript degradation *via* NMD. Quantitative RT-PCR and Western blotting confirmed a meaningful decrease in both WDR26 mRNA and protein levels in these cell lines (Skraban et al., 2017). Clinically, *WDR26* haploinsufficiency causes Skraban–Deardorff syndrome (SKDEAS)—a rare autosomal dominant disorder characterised by global developmental delay, intellectual disability, seizures, ataxia, and dysmorphic facial features (Lin X. et al., 2025).

#### 5.1.3 NAA15 (N-alpha-acetyltransferase 15, NatA auxiliary subunit)

The NAA15 protein is an auxiliary subunit of the NatA N-terminal acetyltransferase complex, which performs co-translational N-terminal acetylation of approximately 40% of all human proteins, including regulators of neurogenesis and synaptogenesis (Dörfel and Lyon, 2015). *NAA15* encodes a component of the NATA complex that presumably anchors it to the ribosome for post-translational protein modification. *NAA15* is highly expressed in the mouse cerebral cortex during periods of proliferation and migration (Tian et al., 2022).

Bioinformatic analysis suggests that 25 Likely Gene-Disrupting (LGD) variants, distributed across the entire length of the *NAA15* gene, are subject to NMD-mediated degradation, leading to the elimination of aberrant mRNA and loss of the protein product. This has been experimentally confirmed: reduced levels of mutant transcripts were identified in a lymphoblastoid cell line from one patient and their near-complete absence in an iPSC line from another. This is likely due to NMD-mediated degradation of the mutant transcript, which results in an approximately 50% reduction in NAA15 protein levels (Cheng et al., 2018). It was also established that a 50% reduction in NAA15 alters the levels of at least 562 proteins (Ward et al., 2021). The clinical presentation includes varying degrees of intellectual disability, speech and motor delays, ASD or behavioural disturbances, as well as mild craniofacial dysmorphisms, congenital heart defects, and seizures in some patients (GÖRKER et al., 2025; Patel R. et al., 2025).

#### 5.1.4 CASK (calcium/calmodulin-dependent serine protein kinase)

The *CASK* gene encodes calcium/calmodulin-dependent serine protein kinase (CASK). CASK is primarily localised in brain neurons, where it controls the expression of genes involved in brain development and regulates neurotransmitter transport and ion exchange necessary for interneuronal signalling (Tabuchi et al., 2026). Beyond its synaptic function, CASK acts as a transcriptional co-regulator *via* TBR1, CINAP (TSPYL2), and BCL11A proteins, all of which are implicated in the pathogenesis of NDD (Becker et al., 2020).

A clinical case demonstrated that the variant c.2506-6 A>G in the *CASK* gene induced two alternative splicing events, accounting for 80% of all transcripts, which were subsequently degraded by NMD (Rodríguez-García et al., 2023). Heterozygous mutations in the X-linked *CASK* gene are associated with intellectual disability, microcephaly, pontocerebellar hypoplasia, optic nerve hypoplasia, and partially penetrant seizures in females (Patel P. A. et al., 2020).

*Cask* ^*+/−*^ female mice exhibit postnatal microcephaly, cerebellar hypoplasia, and optic nerve hypoplasia (Hashemi et al., 2026). Importantly, brain volume changes in CASK-associated disorders result from degenerative cell death rather than impairments in primary development.

CASK-associated disorders manifest in two primary forms: microcephaly with pontocerebellar hypoplasia (MICPCH) and X-linked intellectual disability (XL-ID) with or without nystagmus. Females with the MICPCH subtype typically present with moderate to severe ID, global developmental delay, axial hypotonia, appendicular hypertonia, and movement disorders such as dystonia (Mori et al., 2023). In males, these are associated with early-infantile epileptic encephalopathy—specifically Ohtahara and West syndromes (Martin et al., 2025). Notably, missense variants associated with epilepsy and ID are distributed across the entire length of the CASK protein, whereas missense variants linked to microcephaly and MICPCH are concentrated in specific domains (Silveira et al., 2024). Thus, *CASK* serves as an example of an X-linked gene with dosage sensitivity in neuronal structures. LoF variants, by directing aberrant transcripts for NMD-mediated degradation, reduce protein levels below a critical threshold, disrupting the multiprotein synaptic scaffold and impairing transcriptional regulation of neuronal development. The dosage dependency of the pathogenesis is directly reflected in the clinical spectrum—ranging from severe MICPCH with complete loss of function to mild XL-ID with hypomorphic variants (Zhao J. et al., 2021).

#### 5.1.5 KDM5C (lysine demethylase 5C)

The KDM5C protein is a histone demethylase that catalyses the removal of di- and tri-methylation of histone H3 at lysine 4 [H3K4me2/3]. It is most actively expressed in the brain, where it is critical for dendritic morphogenesis and synaptic plasticity (Jensen et al., 2005).

The gene is located on the X chromosome, which accounts for the more severe phenotype in males compared to females (Brookes et al., 2015). Nonsense and frameshift variants have been described which, by creating a PTC, direct the *KDM5C* transcript for NMD-mediated degradation, thereby reducing functional protein levels to a state of haploinsufficiency (Santos-Rebouças et al., 2011). Direct evidence of NMD degradation was obtained *via* comparative analysis of *KDM5C* mRNA in patients: in a carrier of the frameshift variant c.3223delG, the transcript was undetectable, whereas mRNA was present in patients with missense variants (Brookes et al., 2015). A similar reduction in mRNA levels was experimentally confirmed for nonsense variants c.3864G>A and c.2080C>T using semi-qRT-PCR (Meng et al., 2025). Clinically, *KDM5C* haploinsufficiency causes Claes–Jensen syndrome, which includes mild to severe intellectual disability, autism spectrum disorders, aggression, and epilepsy (Meng et al., 2025).

Additionally, *BRSK2* is considered a candidate gene where LoF variants presumably implement pathogenicity *via* NMD-dependent haploinsufficiency, although direct experimental data are currently limited (Zhang Q. et al., 2025; Hiatt, Thompson, Prokop, Lawlor, Gray, Bebin, Rinne, Kempers, Pfundt, van Bon, et al., 2019). The BRSK2 protein is a brain-specific serine/threonine protein kinase of the AMPK family. In patients with rare heterozygous *BRSK2* variants, developmental delay and/or intellectual disability have been observed (Hiatt, Thompson, Prokop, Lawlor, Gray, Bebin, Rinne, Kempers, Pfundt, van Bon, et al., 2019). Translation of aberrant transcripts with premature stop codons arising from these variants is suppressed *via* the NMD pathway, leading to a reduction in the amount of functional BRSK2 protein to a state of haploinsufficiency (Zhang Q. et al., 2025).

### 5.2 NMD evasion as a phenotype modifier: dominant-negative effects

Section 5.1 examined situations in which NMD functions correctly and degrades the aberrant transcript, causing a reduction in protein levels to a state of haploinsufficiency. However, the outcome of a pathogenic variant depends on exactly where in the transcript the PTC occurs. If the PTC is located in the final exon or fewer than 50–55 nucleotides upstream of the last exon-exon junction, the transcript evades NMD-mediated degradation and is translated into a truncated protein, which may exert a dominant-negative or gain-of-function effect (Hassan et al., 2025; Holbrook et al., 2006). Thus, the fate of the transcript determines the phenotype. The same gene, with a PTC in different positions, can cause two different molecular outcomes and, consequently, two different clinical syndromes.

#### 5.2.1 ADNP (activity-dependent neuroprotective protein)

The ADNP protein is a transcriptional regulator and a component of the ChAHP chromatin-remodelling complex, which plays a critical role in hippocampal development (Carratu et al., 2026). *De novo* pathogenic variants cause Helsmoortel–Van der Aa syndrome (HVDAS), characterised by ID, ASD, and developmental delay (Benvenuto et al., 2026). The severity of the phenotype is determined by the position of the mutation relative to the nuclear localisation signal (NLS). Variants proximal to the C-terminus of the NLS implement a dominant-negative mechanism; variants within the NLS lead to cytoplasmic protein accumulation, while N-terminal variants result in proteasomal degradation of the fragments (D’Incal et al., 2024). The recurrent variant p.Tyr719*, which evades NMD, is associated with the most severe phenotype, manifesting as profound intellectual disability and severe ASD features (Van Dijck et al., 2019). In addition to truncating variants, the missense variant p.C687R, which disrupts the ninth zinc-finger domain, also evades NMD but implements a GOF-like mechanism. It alters the chromatin binding profile and establishes bivalent histone marks H3K4me3/H3K27me3, which leads to abnormal activation of GABAergic lineage genes during neuronal differentiation (Chen Q. et al., 2026). Systemic analysis of PTVesc (Premature Termination Variants escaping NMD) in an NDD cohort confirmed *ADNP* as a gene reaching exome-wide significance for enrichment in NMD-escaping variants (Torene et al., 2024).

#### 5.2.2 ASXL2 (additional sex combs-like 2)

The ASXL2 protein recruits POLYCOMB and TRITHORAX group proteins (involved in embryogenesis), acting as an epigenetic regulator (Minotti et al., 2024). As a non-catalytic subunit of the PR-DUB complex, ASXL2 facilitates the deubiquitination of H2AK119ub1. The complex itself functions as a transcriptional co-activator, regulating genes involved in proliferation, cellular communication, and cell viability (Campagne et al., 2019).

All pathogenic variants associated with the classical form of Shashi–Pena syndrome are *de novo* frameshift, nonsense, or pathogenic splice variants within the final two exons of *ASXL2*, resulting in premature protein truncation (Nakamura et al., 2026). Analysis of patient cells has shown that mutant transcripts are expressed and do not undergo NMD-mediated degradation, indicating a dominant-negative mechanism rather than haploinsufficiency. Thus, the localisation of the PTC within the NMD-refractory zone ensures the stability of the aberrant transcript and the accumulation of a truncated protein lacking C-terminal regulatory domains (Shashi et al., 2016). Clinically, Shashi–Pena syndrome (SHAPNS) is characterised by hypertelorism, a broad nasal tip, arched eyebrows, a V-shaped glabellar nevus flammeus, low-set ears, ptosis, progressive macrocephaly, feeding difficulties, developmental delay, hypotonia, hypoglycaemia, and seizures (Yuan et al., 2023). The syndrome is distinguished from ASXL1-and ASXL3-associated disorders by macrocephaly, the absence of growth retardation, and greater variability in the degree of intellectual impairment (Shashi et al., 2016). Notably, macrocephaly is often progressive, and neurodevelopmental regression is possible in patients with initially normal MRI findings who subsequently develop non-febrile seizures (Yuan et al., 2023).

#### 5.2.3 SHANK1 (SH3 and multiple ankyrin repeat domains protein 1)

The SHANK1 protein is a postsynaptic scaffold protein that organises protein complexes within the postsynaptic density of excitatory synapses (May et al., 2021).

In *in vitro* experiments analysing *de novo* truncating *Shank1* variants, it was determined that the corresponding transcripts are stable and do not undergo NMD-mediated degradation. The truncated Shank1 protein completely loses the ability to bind with the linker protein Homer1, which interacts with the C-terminus of Shank1 (May et al., 2021). Thus, the transcript’s escape from NMD enables the synthesis of a truncated protein lacking the Homer1-interaction domain, which disrupts postsynaptic scaffold organisation. Described patients with truncating variants exhibit a wide spectrum of NDD, including ASD, neuropsychiatric disorders, cognitive delays and/or learning difficulties, motor delays, speech and language delays, and brain anomalies (Manso-Bazus et al., 2025; May et al., 2021).

Additionally, several candidate genes have been identified (NSRP1, POGZ, NSUN6, ZNF496) which are associated with NDD and where a role for NMD-dependent mechanisms in pathogenesis is suggested based on clinical-genetic data and bioinformatic analysis (Calame et al., 2021; Nagy D. et al., 2022; Mattioli et al., 2023; Calì et al., 2025).

Table 2 presents representative examples of genes demonstrating non-canonical scenarios of NMD/AS-NMD involvement in the pathogenesis of NDD. In some cases, the effective degradation of an aberrant transcript results in NMD-mediated haploinsufficiency, whereas in others, evasion of NMD permits the synthesis of a truncated protein with a dominant-negative or gain-of-function effect.

**TABLE 2:** Non-canonical scenarios of pathogenicity mediated by the NMD system in NDD target genes.

Pathogenic mechanisms	Gene	Molecular function	Clinical phenotype
NMD-mediated haploinsufficiency	*ARID1B*	DNA-binding subunit of the BAF chromatin-remodelling complex (Moffat et al., 2022)	Coffin–Siris syndrome; Non-syndromic ID; Microcephaly; ASD (Lian et al., 2020; Moffat et al., 2022; Sofronova et al., 2022)
	*WDR26*	Adapter protein of the CTLH E3 ubiquitin ligase complex (Onea et al., 2025)	Skraban–Deardorff syndrome (SKDEAS); Seizures; Ataxia. Lin X. et al. (2025)
	*NAA15*	Auxiliary subunit of the NatA N-terminal acetyltransferase complex (Dörfel and Lyon, 2015)	ID; ASD; Motor and speech delay. (GÖRKER et al., 2025; Patel R. et al., 2025).
	*CASK*	Calcium/calmodulin-dependent serine protein kinase; transcriptional co-regulator (Tabuchi et al., 2026)	MICPCH syndrome (microcephaly, pontocerebellar hypoplasia); X-linked ID (XL-ID). (Patel P. A. et al., 2020; Mori et al., 2023; Martin et al., 2025)
	*KDM5C*	Histone demethylase (H3K4me2/3) (Jensen et al., 2005)	Claes–Jensen syndrome; ASD; Epilepsy. (Brookes et al., 2015; Meng et al., 2025; Jensen et al., 2005)
Dominant-negative effect (NMD evasion)	*ADNP*	Transcriptional regulator; component of the ChAHP chromatin-remodelling complex (Carratu et al., 2026)	Helsmoortel–Van der Aa syndrome (HVDAS); ID; Severe forms of ASD. (Benvenuto et al., 2026; Van Dijck et al., 2019)
	*ASXL2*	Regulatory subunit of the PR-DUB deubiquitinating complex (Minotti et al., 2024; Campagne et al., 2019)	Shashi–Pena syndrome (SHAPNS); Progressive macrocephaly. (Nakamura et al., 2026; Minotti et al., 2024; Yuan et al., 2023; Shashi et al., 2016)
	*SHANK1*	Postsynaptic scaffold protein (May et al., 2021)	ASD; Cognitive and motor delay; Brain anomalies. (Manso-Bazus et al., 2025; May et al., 2021)

## 6 Conclusion

The data presented in this review provide compelling evidence that NMD and its associated alternative splicing mechanisms play a pivotal role in the pathogenesis of neurodevelopmental disorders. Dysfunction of NMD components is directly linked to the occurrence of intellectual disability, autism, and other NDDs, highlighting the importance of this pathway for the normal development and maturation of the nervous system. Furthermore, aberrant NMD triggered by the inclusion of poison exons can lead to the haploinsufficiency of key genes, contributing to the development of severe conditions such as Coffin–Siris, Skraban–Deardorff, Ohtahara and West syndromes, amongst others.

Diagnostic capabilities have expanded significantly with the recognition that pathogenic variants may affect not only protein sequence but also splicing regulation. The identification of deep intronic mutations that activate cryptic splice sites and trigger NMD enables the establishment of a genetic diagnosis in cases previously classified as non-genetic or idiopathic.

The most promising therapeutic avenue targeted at NMD and splicing regulation is the use of antisense oligonucleotides (ASOs), specifically splice-switching ASOs (SSOs). Their therapeutic effect is based on blocking the inclusion of poison exons, thereby increasing the expression levels of the functional protein. This strategy is capable not merely of symptomatic relief but of addressing the primary cause of neuropsychiatric disorders.

Despite these advances, several critical challenges remain. Currently, our understanding of precisely how poison exons regulate gene expression is incomplete, underscoring the necessity of implementing stage-specific transcriptomics. The expression of alternative splice variants, including poison exons, is tightly regulated both temporally and spatially. For instance, in sodium channel genes (*SCN1A, SCN8A*), the level of poison exon inclusion peaks during prenatal development and infancy, declining sharply as the brain matures. Consequently, the efficacy and safety of ASO therapy may depend critically on the developmental stage and cell type. Dynamic single-cell transcriptomic analysis is essential for accurately defining the therapeutic window for intervention and predicting tissue-specific effects. These therapeutic strategies should be considered a primary focus for future developments in the field, requiring a separate systematic analysis as more data on stage-specific transcriptomics become available.

Further progress in this field will depend on the integration of transcriptomic insights and the development of delivery systems to achieve long-term splicing modification within targeted neuronal populations.
